# Insight into the Antifungal Mechanism of Action of Human RNase N-terminus Derived Peptides

**DOI:** 10.3390/ijms20184558

**Published:** 2019-09-14

**Authors:** Vivian A. Salazar, Javier Arranz-Trullén, Guillem Prats-Ejarque, Marc Torrent, David Andreu, David Pulido, Ester Boix

**Affiliations:** 1 Department of Biochemistry and Molecular Biology, Faculty of Biosciences, Universitat Autònoma de Barcelona, 08193 Cerdanyola del Vallès, Spain; javiat7891@gmail.com (J.A.-T.); Guillem.Prats.Ejarque@uab.cat (G.P.-E.); Marc.Torrent@uab.es (M.T.); david.pulido-gomez@ndm.ox.ac.uk (D.P.); 2 Department of Experimental and Health Sciences, Universitat Pompeu Fabra, Dr. Aiguader 88, 08003 Barcelona, Spain; david.andreu@upf.edu

**Keywords:** antimicrobial peptides, *Candida albicans*, biofilms, antifungal activity, RNaseA superfamily

## Abstract

*Candida albicans* is a polymorphic fungus responsible for mucosal and skin infections. *Candida* cells establish themselves into biofilm communities resistant to most currently available antifungal agents. An increase of severe infections ensuing in fungal septic shock in elderly or immunosuppressed patients, along with the emergence of drug-resistant strains, urge the need for the development of alternative antifungal agents. In the search for novel antifungal drugs our laboratory demonstrated that two human ribonucleases from the vertebrate-specific RNaseA superfamily, hRNase3 and hRNase7, display a high anticandidal activity. In a previous work, we proved that the N-terminal region of the RNases was sufficient to reproduce most of the parental protein bactericidal activity. Next, we explored their potency against a fungal pathogen. Here, we have tested the N-terminal derived peptides that correspond to the eight human canonical RNases (RN1-8) against planktonic cells and biofilms of *C. albicans*. RN3 and RN7 peptides displayed the most potent inhibitory effect with a mechanism of action characterized by cell-wall binding, membrane permeabilization and biofilm eradication activities. Both peptides are able to eradicate planktonic and sessile cells, and to alter their gene expression, reinforcing its role as a lead candidate to develop novel antifungal and antibiofilm therapies.

## 1. Introduction

*Candida albicans* is the most common fungal pathogen that threatens hospitalized and immunocompromised patients. Together with candidiasis in skin and mucosal infections, fungal septic shock cases are also reported in elderly or immunosuppressed patients [1]. Most of the serious symptoms of candidiasis are associated with biofilm formation occurring on the surfaces of host tissues and medical devices, enhancing *C. albicans* virulence and resistance. Moreover, the most representative feature of *Candida* biofilms is its increasing tolerance to conventional antifungal therapy. The protection of the biofilm embedded cells against noxious agents, such as antifungals and the host immune system arsenal, favors the thriving of persister cells and hinders the treatment of biofilm-associated infections [2,3]. It has been estimated that the eradication of biofilm communities requires concentrations of antifungals 1000 times higher compared to their planktonic stage [4,5]. This fact drastically reduces the availability of antifungal drugs appropriate to tackle *C. albicans* biofilms. Frequently, drug doses need to be increased over the safety level [2]. Moreover, we are currently facing an increase in yeast pathogenesis along with the emergence of drug-resistant strains, urging the need for innovation of new effective antifungal agents. Antimicrobial proteins and peptides (AMPs) are considered one of the main ancestral host defense systems. Largely distributed throughout the different organism tissues, AMPs play an essential role as part of the human innate immune system, constantly protecting the body against microbial invasion and diseases [6,7,8]. Because of their wide distribution, physicochemical features, biological activities and rapid antimicrobial action against a broad spectrum of microbes, AMPs have recently attracted significant attention as encouraging antibiotic candidates [9,10,11,12,13]. Furthermore, due to their direct action at the microbial membrane and multifaceted intracellular killing mechanisms, the use of AMPs as antibiotics should reduce the appearance of resistant pathogens, a scenario that has been favored by the abuse of conventional antibiotics [14,15]. The emergence of resistance mechanisms associated with fungal infections has also encouraged the characterization of natural host defense peptides as lead candidates for the design of novel drugs. In particular, several synthetic AMPs were successfully tested against *C. albicans* [16]. Among the human peptides that participate in the host innate immunity against fungal infections, we find cathelicidins, histatins and defensins, that combine direct action to the pathogen cell together with indirect immune-modulation activities [17]. 

In our research group, we have explored the use of human secretory RNases as antimicrobial agents against Gram-negative and Gram-positive bacteria [18]. Following structure-functional studies, an RNase derived peptide was proposed as a novel antibiofilm agents against Gram-negative bacteria [19,20]. Likewise, in the search for novel antifungal agents, we recently reported that human antimicrobial RNases are also effective against *C. albicans* through a dual mode of action [21,22]. Interestingly, the fungicidal activity of the RNases is initiated by the binding to the pathogen cell wall, followed by their translocation and intracellular targeting [21]. In previous attempts to define the antimicrobial activity of the hRNases, we designed the derived peptides corresponding to the N-terminal of all them. The results demonstrated that the main antimicrobial activities are retained at the first 45 residues of the parental ribonucleases. A comparison between each N-terminal derived peptide and the related parental protein confirmed that their abilities to disrupt model membranes and inherent antimicrobial activities were retained [23,24,25]. In this work, we have explored the mechanism of action of the 1–45 N-terminus peptides of human canonical RNases (hRNases) against *Candida* planktonic cells and the action toward established and early-biofilms. The promising results place hRNase-derived peptides in the spotlight as therapeutic lead compounds and reinforce the value of our own innate immune response arsenal in the fight against fungal infections. 

## 2. Results

### 2.1. Peptides Design and Physicochemical Characterization

**Figure 1:**
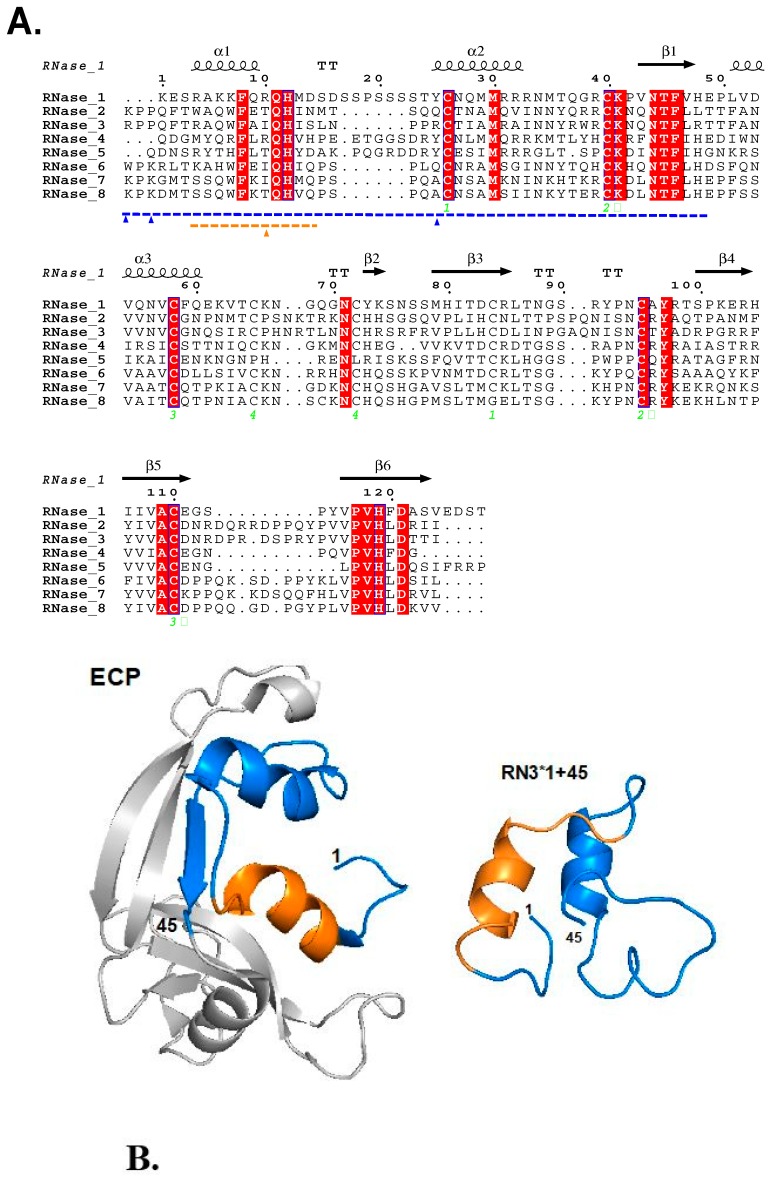
(**A**) Sequence alignment of hRNases. Conserved regions are boxed; highly conserved amino acids are colored in white over a red background, whereas moderately conserved amino acids regions are colored in red. The secondary structure of hRNase3 is displayed as a reference on top of the alignment. The N-terminal antimicrobial domain is highlighted in blue and the agglutination-promoting region in orange. (**B**) The tertiary structure of Eosinophil cationic protein (ECP), the N-terminal domain is colored according to the antimicrobial (blue) and aggregation (orange) properties (PDB code 4A2O). Right hand: Model representation of RN3 1–45, the figure was performed by PEP-FOLD3 (http://mobyle.rpbs.univ-paris-diderot.fr/cgi-bin/portal.py#forms::PEP-FOLD3).

The hRNase-derived peptides were designed, taking as reference the 1–45 segment in hRNase3 sequence, based on previous work in our laboratory that defined the protein minimal domain retaining full antimicrobial properties against Gram-positive and –negative bacteria. The N-terminal residues of the hRNases represent a conserved evolutionary region that retains the protein host-defense properties [23,24,25]. Figure 1 and Table 1 summarizes the main physiochemical, structural and biological properties of the selected peptides. The eight N-terminal peptides (RN1–8), comprising equivalent structural regions of the human canonical RNases N-termini [residues 1–45 of hRNases 2, 6, 7 and 8; residues 1–48 of hRNases 1 and 4; and residues 1–47 of hRNase 5;] were selected for synthesis to test their antimicrobial capabilities against *C. albicans*. In all cases, the peptides included the first two α-helices, as well as the first β-strand from the parental protein. As previously, the two cysteine residues present at the N-terminal region were replaced by isosteric serine residues [23,25]. Previous structural analysis and predictive studies indicated that the peptides adopted an helix in a membrane-like environment [23] (Figure 1 and Figure S1). Complementarily, Lys/Arg RNase N-terminal derived peptides were engineered for the two most active peptides (RN3 and RN7), which incorporate Arg to Lys substitutions for RNase 3, and Lys to Arg substitutions for RNase 7, named as RN3K and RN7R respectively [23]. The substitution of Arg versus Lys in peptide structures sought to increase the cell penetration capacity, as reported previously [26,27]. It is noteworthy to underline the high pI and degree of hydrophobicity of the peptides (Table 1), adopting an amphipathic structure (Figure S1) and showing values within the standard range of most-known AMPs [28], which are considered essential for their functional activity [29]. Table 1 also includes the relative estimated values for the antimicrobial activity of the hRNase parental proteins, as referenced in our previous work [30]. 

### 2.2. Antifungal Activity against Planktonic Cells of hRNases Is Retained at the N-terminus 

Previous work in our laboratory using *C. albicans* demonstrated a high antifungal activity for hRNases 3 and 7 [21]. Here, we report the antifungal activity of the eight N-terminal peptides corresponding to the eight human canonical RNases. First, the minimum fungicidal concentration (MFC) and 50% inhibitory concentration against infective *C. albicans* was determined. MFC values are summarized in Table 2. Five peptides (1, 3, 4, 6 and 7) were found active against *C. albicans*, with MFC ranging from 0.6 to 4 µM, a range close to that observed for the previously tested hRNases [21]. On the other hand, peptides 2, 5 and 8 were inactive. A comparison of the bactericidal and candidacidal activities of hRNases (Table 1 and Table 2) highlighted a positive correlation, confirming that the antimicrobial activity is preserved at the N–terminus. 

On the other hand, no significant cytotoxicity for human THP1 cells is observed for the peptides at the highest tested concentration (50 μM) (Table 1). Likewise, previous characterization of the peptide hemolytic activities [23] also confirmed that no significant toxicity is observed at more than 10x the achieved fungicidal concentration. The peptides RN3K and RN7R were also tested in their antifungal activities, but the concentrations to reach an antimicrobial activity were higher in relation to their wild type peptide (Supplementary Table S1). In the same way, no differences were observed for the respective cytotoxic activity of either Lys/Arg peptide variants on host cells (Table 1).

Another specific feature of the mechanism of action of some AMPs is the ability to agglutinate bacteria, a property proposed to facilitate the removal of the infection focus. Considering that hRNase3 and its N-terminal domain [31] displayed a significant bacterial agglutinating activity, we also assayed the RNases 3 and 7 action on *Candida* cell cultures. Accordingly, we confirmed that hRNase3 is able to agglutinate *C. albicans* but not hRNase7 when tested up to 20 μM, in accordance with their respective bacterial and liposome agglutination activities [31,32]. Following up, in order to check whether hRNase-derived peptides also share this property against *C. albicans*, their minimum agglutinating activity (MAC) was determined. Surprisingly, all peptides showed cell agglutination activity (MAC < 2 µM), indicating that the required features for yeast cells agglutination are shared by all the hRNases N-terminus (see supplementary Table S2 and Figure S2). In any case, after incubation of *C. albicans* cell cultures with the hRNase-derived peptides, the most significant agglutination was observed for RN3 (<0.1 µM), as previously observed for Gram-negative bacteria [23]. In addition, when comparing MAC results between *C. albicans* and *E. coli*, we also observed a positive correlation for peptides RN3 and RN6, being the two more agglutinating peptides within the family [23]. Interestingly, a comparison of the MAC values indicate a higher agglutinating activity for *C. albicans* in comparison to bacteria, suggesting that the peptides should have a particularly high affinity for fungi wall components. In previous works, we assayed hRNase3 interaction to different bacterial cell wall components such as lipopolysaccharide (LPS) and peptidoglycans [32,33], as well as heterosaccharides present at the eukaryote extracellular matrix [34,35,36]. Here, we have characterized the peptides’ affinity for the fungi cell wall and associated mechanism of action. 

### 2.3. The Antifungal Mechanism of hRNase-Derived Peptides Relies on Membrane and Cell Wall Interactions

As reported in our previous work on antimicrobial hRNases, one of the first steps to counteract the bacterial growth relies on the protein cell wall binding and membrane destabilization activity [31,33]. In an effort to characterize the antifungal mechanism of hRNase-derived peptides (RN1-8), we assessed their capacity to depolarize and permeabilize *C. albicans* cytoplasmic membrane. Results showed that overall most of the peptides active against *C. albicans* displayed high membrane permeabilization and depolarization values (Table 3). The data agrees with previous depolarization and leakage values in both Gram-negative and Gram-positive bacteria species. Noteworthy, RN3, RN6 and RN7 peptides displayed 50% Effective Displacement (ED_50_)depolarization values about 0.5-0.6 μM, with a maximum depolarization value at final incubation time for RN3. In relation to *Candida* membrane permeabilization and percentage of cell survival, the same three peptides showed the most effective permeabilization, with mortality percentages above 70% after 2 hours of cell culture incubation with the peptides. For a better characterization of the mechanism, we compared the kinetics of the membrane destabilization and killing process by hRNase-derived peptides (Table 3). Working at a selected peptide concentration around the estimated MFC values (2 μM), the time to reach 50% effectivity was estimated. RN3 showed the best values in terms of cell survival, depolarization and permeabilization, followed by RN7 and RN6. Previously, RNase derived N-terminal peptides were reported to have the ability to bind anionic liposomes and trigger the vesicles lysis [23]. Moreover, as it has been reported for other AMPs [37], a positive correlation had been observed regarding the lytic activity of the parental RNases in both synthetic lipid vesicles and bacterial cells [31,32,38]. The present study on *C. albicans* cell culture corroborates that these peptides share the characteristic AMPs features, displaying an unspecific antimicrobial activity against both prokaryotic [23] and eukaryotic pathogens.

**Figure 2:**
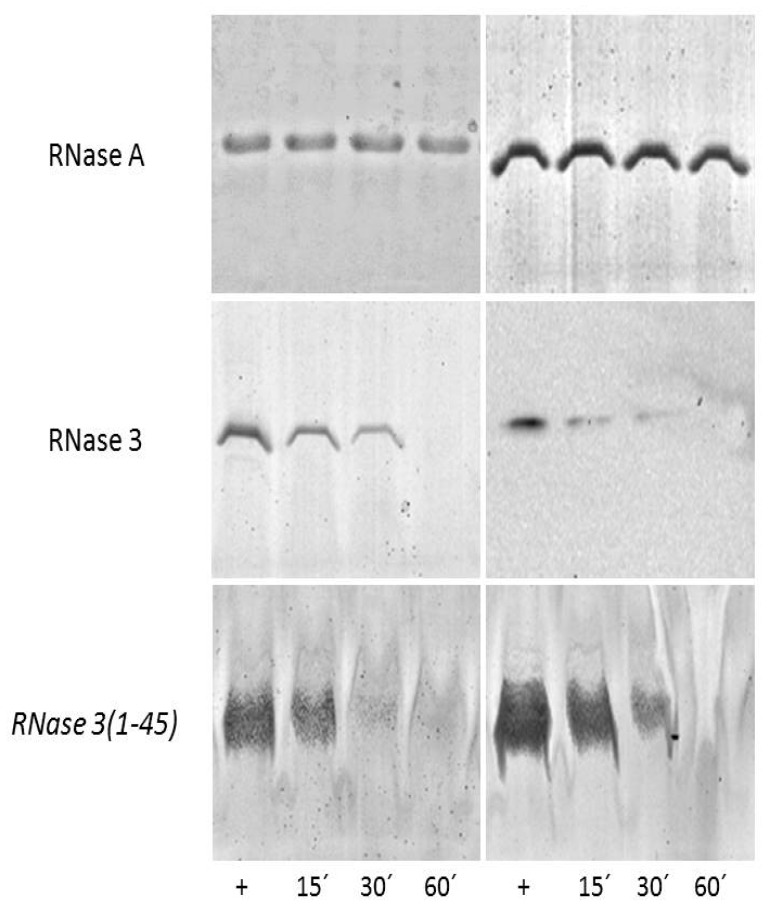
Binding of hRNase3 peptide to *C. albicans* cells (left panels) and β-d-Glucans (right panels). RNase A was taken as a negative control for cell and β-d-Glucan binding, respectively. *C. albicans* cells and β-d-Glucan were incubated at different time (15, 30 and 60 min) with the protein and RN3 peptide: hRNase3(1–45). The supernatant soluble fractions were prepared as described in the Experimental Procedures. Supernatant represents the soluble fraction, which contains the unbound protein/peptide. 15% and 18% SDS-PAGE were respectively used to separate the protein and peptide fractions. Protein and peptide alone were also loaded to the gel as positive controls (+).

On the other hand, cationic peptides can display a high binding affinity toward anionic polymeric structures, such as the bacterial and fungal cell envelopes. Two main layers can be distinguished in the *C. albicans* cell wall: an outer layer composed mainly of glycoproteins and an inner layer in which polysaccharides predominate. These polysaccharides confer strength and favor the cell shape. Beta-1,3 glucan, β-1,6 glucan and chitin are the most common polysaccharides, comprising 40%, 20% and 2% of the cell wall dry weight respectively [39]. In particular, β-1,3-Glucan, the major component of the fungi cell wall, is also a key element of the biofilm extracellular matrix [40,41]. Here, we selected hRNase3 and its N-terminal derived peptide as the most antimicrobial active member of the family, to assess the binding ability to both *C. albicans* cells and the β-d-Glucan (linear β-1,3-d-Glucan and branched d-Glucose 1,6 units). Results shown in Figure 2 confirm that hRNase3 and the RN3 peptide are able to bind to the *C. albicans* cell wall, and that the binding percentage increases as a function of the incubation time.

### 2.4. Nucleic Acids Binding of RN3 and RN7 the Potent Anticandidal Peptides

**Figure 3:**
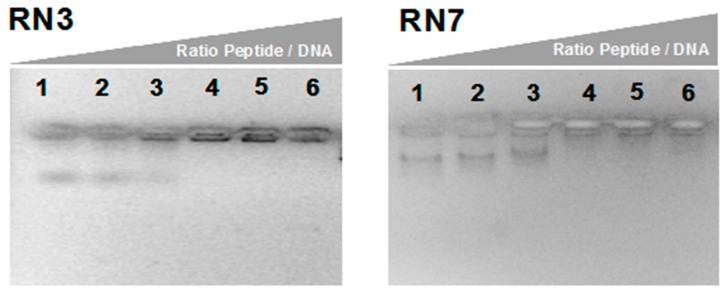
Gel retardation assay. Binding DNA was assayed by the inhibitory effect of peptides on migration of DNA. Different amounts of peptides were incubated with 200 ng of pET 28a plasmid DNA in 10 µL of binding buffer at room temperature during 20 min and subjected to electrophoresis on a 1.0% agarose gel. The first lane corresponds to negative control without peptide and the following lanes correspond to weight ratios peptide/DNA 2.5:1, 5:1, 10:1, 20:1 and 50:1 respectively.

Considering our promising results obtained with the characterization of the peptides’ antifungal activities and cell- wall binding abilities, we decided to analyze the action of the two most potent antifungal peptides (RN3 and RN7) in their abilities to bind DNA. We also based our selection in the physicochemical properties of these peptides that could facilitate the nucleic acid binding ability. Our previous work has demonstrated the effective internalization of hRNases3 and 7 within *C. albicans* cells [20]. Here, we have tested the peptides’ affinity to nucleic acids, as putative intracellular targets. The in-vitro DNA binding ability was determined by monitoring the electrophoretic mobility of plasmidic DNA on an agarose gel. Results indicated that RN3 and RN7 bind to the plasmid DNA and retarded its gel migration in a concentration-dependent manner (Figure 3). Similar affinities for DNA were observed for the RN3K and RN7R counterparts (Supplementary Figure S3). This fact encouraged us to continue the exploration of the potential action of these peptides over *Candida albicans’* expression pattern. 

### 2.5. Evaluation of the Activities of RN3 and RN7 Peptides against C. albicans Biofilms 

**Figure 4:**
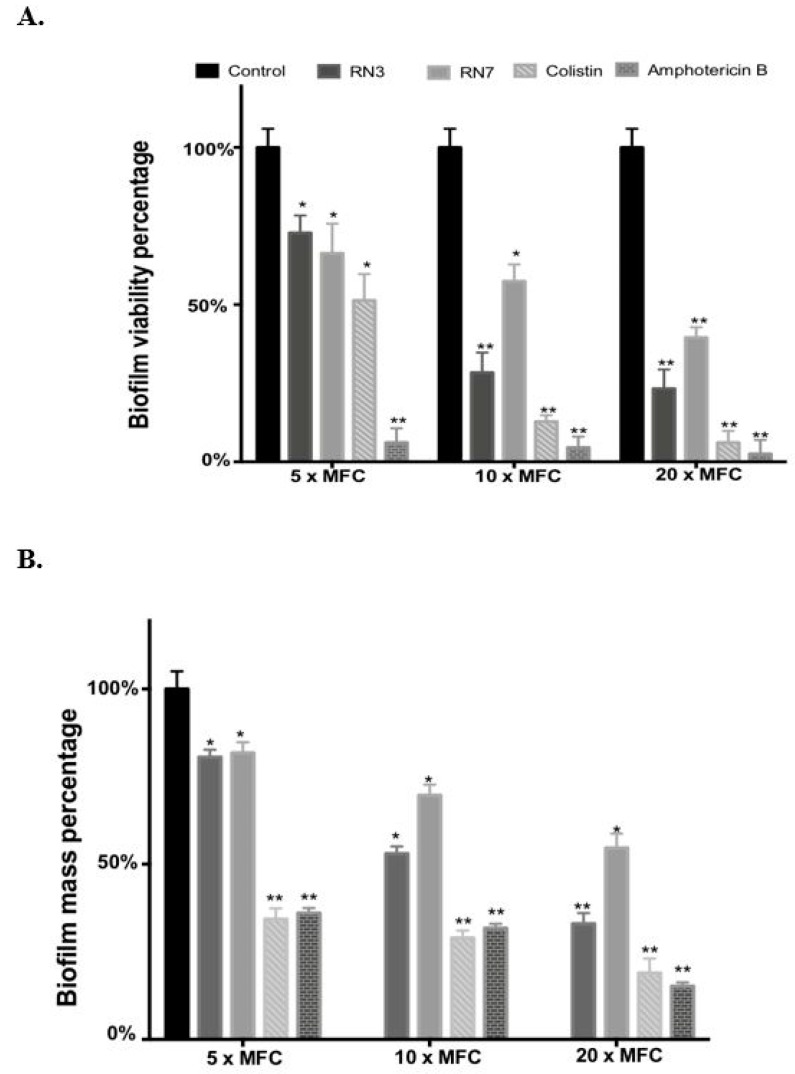
Effect of peptides (RN3 and RN7), colistin and amphotericin B on stablished *C. albicans* biofilms. (**A**). The viability of preformed biofilms was quantified by *PrestoBlue* dye after 24 h of incubation with the corresponding treatment at 5×, 10× and 20× MFC (Peptide concentration 5, 10 and 20 μM; amphotericin B 2.5, 5 and 10 μM and colistin 1.25, 2.5 and 5 μM). Fluorescence changes were measured at excitation/emission of 535-615 nm. (**B**) The biofilm was stained by crystal violet, then the dye was solubilized in ethanol, its absorbance measured at 570 nm and the percentage of biofilm reduction was compared to untreated wells control. All experiments were done in triplicate. Statistically significant results were evaluated by ANOVA test, followed by Dunnett’s Multiple Comparison Test (* *p* < 0.05 and ** *p* < 0.001).

In order to get further insight into the mechanism of action of RN3 and RN7 derived peptides against *C. albicans*, we evaluate the efficacies of these peptides to eradicate pre-established biofilms. We measured the viability and the biofilm mass following peptide treatment by resazurin reduction and crystal violet staining. Additionally, we evaluated by confocal microscopy the biofilm viability and overall structure. Biofilms formation is often associated with antifungal resistance, as compared to planktonic cells, and requires drug concentrations of about 30–2000 times to achieve equivalent reduction of the cell metabolic activity [42]. Here, we assayed the peptide concentrations corresponding to 5, 10 and 20 x MFC fold. For RN3 and RN7 peptides, an average MFC value of 1 μM was taken as a reference. For amphotericin B and colistin we took as an internal reference MFC values of 0.5 and 0.25 μM, respectively; as previously reported [43,44,45]. The antibiofilm activity of the hRNase-derived peptides was assessed using PrestoBlue^TM^ reduction assay, which enables quantitation of the number of living cells in 24h-old biofilms after treatment. The antibiofilm activity of all peptides was compared at an equivalent MFC fold concentration. As observed in Figure 4, RN3 and RN7 peptides significantly reduced *C. albicans* viability and mass of biofilms cells, compared to results with the non-treated cells. An effective reduction of the biofilm viability is already significant at 5 μM for both RN3 and RN7 peptides. The biofilm viability decreased to about 30% for the RN3 peptide at a concentration of 10 μM, highlighting potent action against of the pre-established biofilms of *C. albicans* (Figure 4).

**Figure 5:**
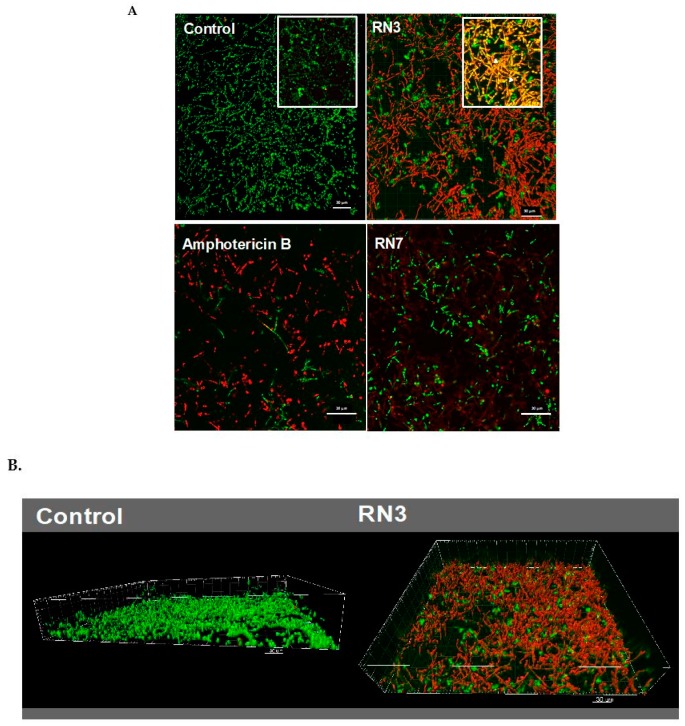
(**A**) Visualization by confocal microscopy of mature *C. albicans* biofilms treated with 20 μM of RN3, RN7 and amphotericin B. Live/Dead stain corresponds to green and red labelled cells, respectively. Right-up detailed zone in control and RN3 images correspond to the overlapping of SYTO9 and PI, showing an increment of hypha formation and reduction of hyphal-viability. (**B**) 3D reconstruction and projections using the IMARIS software^®^ of control and treated biofilm with RN3 peptide. The percentage of viability in the biofilm was reduced to 18% ± 5. Bars: 30 μm.

Interestingly, RN3 peptide showed a similar activity to the one displayed by colistin. However, the RN3 peptide did not achieve the antibiofilm activity values registered for amphotericin B, which is currently considered one of the most effective antifungal agents [44]. Unfortunately, the main problem encountered in the clinical use of amphotericin B is its high cytotoxicity and limited pharmacological application, due to its adverse side-effects, such as fever, vomiting, anemia and nephrotoxicity [46]. To visualize the antibiofilm action of RN3 and RN7 peptides, the pre-established biofilms were incubated during 4h with 20 µM of each peptide. Amphotericin B was used as a control of inhibition. Quantitative analysis by confocal microscopy was performed using Live/Dead staining to estimate the percentage of cell mortality of the biofilm total population and generate a 3D representation of the layer depth. Both peptides drastically reduced the *C. albicans* viability to about 20%, as quantified by PI staining (Figure 5). Interestingly, the samples treated with the peptides, and RN3 in particular, showed a morphological change from yeast to hyphae stage that was not observed in the control biofilm (Figure 5). The untreated biofilm control was composed of non-branched pseudo and yeast forms. We can hypothesize that as a mechanism of defense against the anticandidal treatment, the sessile cells within the biofilm try to evade the antimitotic action by hyphae formation, limiting thereby the drug penetration into the biofilm.

### 2.6. Modulation of the Gene Expression Profile Related to Cell Wall Synthesis and Biofilm Formation in Planktonic Cells 

**Figure 6:**
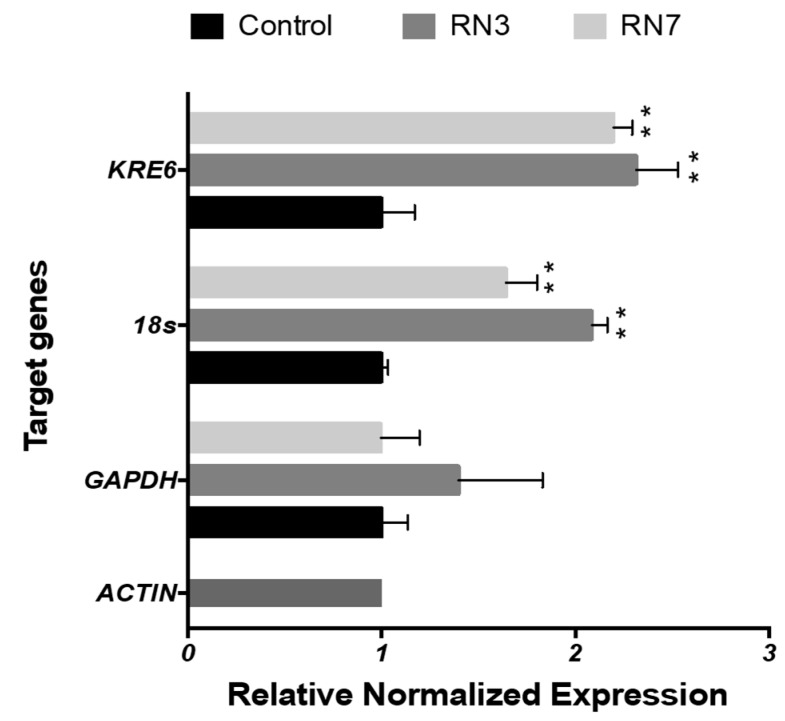
Gene expression profile in planktonic cells of *C. albicans* cells after treatment with 0.5 μM of RN3 and RN7 during 120 min of incubation. The mean values are averaged from two independent experiments performed in duplicates. Statistically significant results were evaluated by ANOVA test, followed by Dunnett’s Multiple Comparison Test. Significant values are indicated respect to the non-treated cells (** *p* < 0.001); all the values are normalized in respect to ACTIN expression.

Following up, we investigated whether the peptide treatment could alter the gene expression profile of *Candida* cells. First, we evaluated by qRT-PCR selected genes related to common metabolic activities as protein synthesis, glycolysis or synthesis of β-glycan wall-component using ACTIN as a housekeeping gene. The first approach to determinate the changes in the gene expression was quantified in *Candida* planktonic cells using a peptide concentration of 0.5 μM during two hours of incubation, at the same time the cell viability was monitored by ATP quantification in order to maintain the cellular population at equal proportions (control and treated cells). The results showed up-regulation of 18s and KRE6 expression after incubation with both RN3 and RN7 peptides (Figure 6). High expression of 18s might respond to a cellular stress caused by the peptides addition. Interestingly, the increase in the *KRE6* expression, a gene that encodes for the synthesis of the cell wall β-glycan, might reflect a defense mechanism against the peptide action.

**Figure 7:**
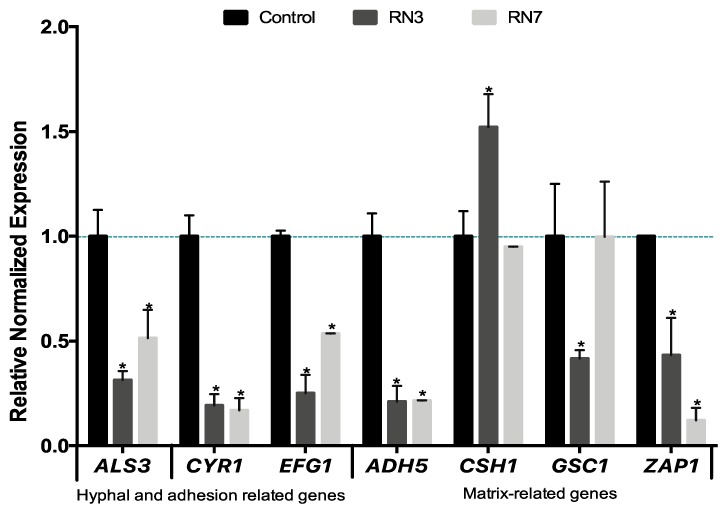
Quantitative RT-PCR analysis of *C. albicans* biofilm-related genes after 10 μM of RN3 and RN7 treatment. *C. albicans* biofilms, at early stage of development (90 min), were incubated in the presence or absence of both hRNase-derived peptides during 24 h, and then assessed by qPCR analysis. The relative levels of gene expression are presented as fold changes in peptide-treated groups (RN3 peptide in dark grey and RN7 peptide in light grey) with respect to the untreated controls in black. All gene transcript levels were normalized against *ACT1* gene expression (pointed line). Statistically significant results were evaluated by ANOVA test, followed by Dunnett’s Multiple Comparison Test. * *p* < 0.001 indicates significant differences between peptide- treated and control groups.

Lastly, we evaluated the effect of the RN3 and RN7 peptides on the expression of some genes related to the biofilm formation. The potential molecular mechanism behind the ability of these RNase derived peptides to prevent the growth of *C. albicans* biofilms was studied in a culture of sessile cells exposed to 10 μM of each peptide during 24 h of incubation (Figure 7). We selected genes involved in the early stage of hyphal formation, biofilm adhesion process (*ALS3*), expression of hyphal transcriptional regulators in the cAMP-dependent protein kinase pathway (*CYR1* and *EFG1*) and production of the extracellular matrix (*ADH5, CSH1, GSC1* and *ZAP1*). Following the incubation of *C. albicans* cultures with both peptides we observed the down-regulation in many genes related to the biofilm formation. Our results demonstrated suppression in the expression level of the hyphae-specific gene that encodes for surface adhesins (*ALS3*) and the *CYR1* and *EFG1* transcriptional regulation genes. *ALS3* is a downstream component of the cAMP-PKA pathway and is positively regulated by *EFG1*. Finally, the expression levels of genes involved in the extracellular matrix production were also modulated following both peptides’ incubation. After RN3 incubation, the expression level of *ADH5, GSC1* and *ZAP1* were down-regulated, with the exception of the *CSH1* gene, which was up-regulated. A similar pattern was displayed after incubation with RN7, except for the *GSC1* and *CSH1* genes, that did not show any significant change. The results suggest that both RN3 and RN7 peptides inhibition of the growth of *C. albicans* is partly mediated by the cAMP dependent kinase signaling pathway although future work would be necessary to identify the specific altered pathways. The results also indicate that the hRNase derived peptides treatment can also affect the biofilm formation by reducing the levels of adhesion proteins, interfering with hyphae formation and hindering the proper production of extracellular matrix components, which are essential for biofilm formation. 

## 3. Discussion

A high number of antimicrobial peptides have been proposed as alternative antibiotics to combat microbial resistance. In contrast, there is still a scarce availability of peptide-based antifungal drugs [2,47] and only very few are in clinical trial [11,48,49]. The virulence of *C. albicans* is mostly associated with its ability to adhere to the host exposed surfaces and establish organized biofilms communities, which are hard to eradicate. Noteworthy, Andes and coworkers observed the presence of host proteins within the extracellular matrix of fungal biofilms, with an abundance of leukocyte byproducts [50]. Indeed, proteins from our innate immune system embody the principal source of lead candidates to develop novel antimicrobial agents. Within this context, our laboratory has explored the antimicrobial mechanisms of action of the hRNases secreted by innate cells [22,51,52]. Several members of the vertebrate-specific RNase A superfamily have shown antimicrobial features apparently unrelated to their catalytic activity [30,53,54]. In an attempt to deepen the understanding of their biological role it has been proposed that RNases might have emerged as a host-defense family in vertebrate evolution [22,55,56].^.^ Our previous studies on RNase A family members have revealed that the antimicrobial activity is retained by their N-terminal-derived peptides [19,20,23,24]. In particular, the action of the N-terminus of hRNase3 was characterized against Gram-positive and Gram-negative bacteria. The protein 1-45 segment includes the first two α-helices from the parental protein, and NMR studies indicate that the in-solution peptide adopts a unique extended alpha-helix [25,34] (Figure 1B and Supplementary Figure S1). Figure 1B illustrates the helices α1 and α2 of the native ECP and the key residues implicated in antimicrobial and aggregation activities. Complementarily, Supplementary Figure S1 shows how the helical secondary structure would provide to the N-termini peptide a characteristic amphipathic nature. A comparative evolutionary study across vertebrate RNases lineages suggested that the N-terminal region has been conserved to carry out a host defense function [23]. Structural comparison of the structure of the hRNase3 N-terminus solved by NMR [34] and the comparative CD analysis of the eight hRNases respective peptides indicated that without sharing a high sequence identity, all the peptides adopt an equivalent structure [23]. Noteworthy, as reported for many other AMPs, the eight peptides were mostly unstructured when free in an aqueous solution and increased their alpha-helical content in the presence of a membrane like environment [23]. 

In order to expand knowledge of the mechanism of action of hRNases against *C. albicans*, which was observed to take place by a dual mode [21], we aimed here to characterize the antifungal properties of the hRNase derived N-terminal peptides. This comparative study of the N-terminal region of the eight human hRNases against an eukaryotic pathogen such as *C. albicans,* has allowed us to deepen in the mechanism of action of these proteins. The present results corroborate that the antimicrobial activity of hRNases against fungi is also preserved at their N-terminal domain. The data reveal a high antifungal activity, with MFC values at sub micromolar range for most tested derivative peptides, being the RNase N-terminal derived peptides 3, 6 and 7 the most effective ones (Table 2). These results are in agreement with previous studies where the parental proteins were tested against *C. albicans* [21] and the derivative peptides assayed against different Gram-negative/Gram-positive bacterial species [23,24,32,57]. In addition, the current data using *C. albicans* reinforce the correlation between the peptide antimicrobial mechanism of action and the respective depolarization and membrane permeabilization activities displayed by the eight parental RNase peptides, in agreement with previous results [23].

Another specific feature of the N-terminal peptides is the cell agglutinating ability, proposed as a mechanism to restrain the pathogens at the infection focus [54,58]. Previous studies highlighted the bacterial agglutination for both hRNase3 and its N-terminal peptide [23,54,59]. Interestingly, higher agglutination activities are observed for *Candida* cells in comparison to the tested Gram-negative bacteria [23,59]. In particular, an outstanding high activity is observed here for the RN3 peptide. Previous work identified aggregation prone domains at the N-terminus of RNases, showing a correlation between positive agglutination values and the sequences aggregation propensity [23,54]. Besides, the bacteria cell agglutination was also observed to be enhanced by the LPS interaction [58]. We report here positive binding of hRNase3 and its derived peptide for both *Candida* cells and their main cell wall component (Figure 2). Further work is to be carried out to evaluate the distinct binding affinities toward the fungi cell wall patterns. 

Additionally, peptides corresponding to Arg to Lys and Lys to Arg substitutions for the N-terminal RNase 3 and 7 homologues were evaluated in order to check the contribution of Arg/Lys enrichment in the peptide antipathogen selectivity (Figure 1). There is evidence in the literature about the importance of some residues that favors endocytosis. It has been suggested that the quantity of Arg and Lys residues, as well as their ratio and distribution, are essential in some AMPs and cell penetrating peptides (CPPs) for cellular uptake [60]. Our previous work on hRNases antifungal activity demonstrated the ability of hRNases 3 and 7 to internalize within *C. albicans* cells [21]. Interestingly, Arg content in peptides is reported to facilitate the mammalian cellular uptake and improve their cytotoxic activity. However, not only the cationic residue composition of peptides was found to be imperative, but also the primary and secondary structures of the peptides have a high impact in the effectiveness of AMPs to cross eukaryotic membranes and target intracellular dwelling pathogens [61]. In our case, the RNases3 and 7 Arg/Lys analogs (RN3K and RN7R) were less effective against *C. albicans* than their respective reference peptides (Supplementary Table S1). On the other hand, no significant changes were observed when the analog peptides were assayed in Gram-negative and Gram-positive bacterial cells [23]. 

Cationic antimicrobial peptides have attracted interest due to their high binding affinity to cell anionic polymers, and nucleic acids in particular [62,63,64]. Interestingly, our results showed that the most active antimicrobial peptides, RN3 and RN7, have a high binding affinity to DNA. However, even though, the RN3K and RN7R peptides retain or even increase the positive net charge (Table 1), their DNA binding affinity is not enhanced (Figure 3 and Supplementary Figure S3). Likewise, as commented above, no improvement of the antifungal activity of the peptide variants was observed (Supplementary Table S2). Therefore, our data indicate that other structural determinants of the peptides are required for their optimal overall performance. In any case, no significant alteration is predicted for the overall helical content and nucleotide binding regions of Lys and Arg peptide variants (Supplementary Figure S4).

To complement this work, we have explored the peptides’ ability to alter the *C. albicans* gene expression profile. First, we monitored the expression pattern of selected representative genes related to the overall cell metabolism and synthesis of cell wall components (*GAPDH, 18s* and *KRE6)* in planktonic cells after peptide incubation for two hours. Interestingly, we observed an up-regulation of the *18s* subunit and *KRE6* gene expression (Figure 6). Our results are in accordance with studies reporting changes in the profile expression of *C. albicans* in response to other AMPs, such as LL-37 or MAF-1A peptides [65]. The authors observed an induced expression of genes related to the *Candida* cell wall synthesis and anti-oxidative stress [66]. The up-regulation on the expression level of *KRE6* demonstrates a possible activation of the polysaccharide synthesis pathway to maintain cell wall integrity and morphogenesis after the incubation with RN3 and RN7 (Figure 6), as reported previously for other antimicrobial peptides [65]. One putative target of hRNase peptides might be a main cell wall component, such as the β-glucan polymer. Therefore, we decided to explore whether the RN3 and RN7 peptides could halt the growth and formation of *Candida* biofilms, as has been reported for other AMPs [67,68].

It is worth stressing that growth of *C. albicans* biofilm communities within intravenous lines and medical devices poses a serious clinical challenge. In particular, a high resistance to common antibiotics is reinforced by the presence of quorum-sensing molecules that play an important role in the biofilm formation, the release of virulence factors and the protection conferred by the extracellular matrix [5,42,69]. Unfortunately, despite the advances in antimicrobial therapy, *Candida* biofilms remain a challenge because biofilm embedded cells are tolerant towards most conventional antimycotics and there are only few novel agents that can be used to treat biofilm-related infections. To date, only miconazole, caspofungin, anidulafungin and liposomal formulations of amphotericin B are used to effectively treat these infections [44,45]. However, due to serious side effects [46,70] there is a need to identify novel antibiofilm compounds. Besides, current antifungal drugs have few specific targets (i.e., ergosterol biosynthesis and 1,3-β-d-glucan synthesis), and it is widely agreed that antifungal drugs with new mechanisms of action are needed [71]. An appropriate alternative relies on cationic peptides, which establish electrostatic interactions to anionic phospholipids in fungal membranes, such as phosphatidylserine and phosphatidylinositol, or to wall components, such as mannoproteins [72,73,74]. We find seldom examples of AMPs with antifungal activity, such as LL37, histatin5, the N-terminus of human lactoferrin or the KP killer peptide [75,76,77]. Frequently, the antimicrobial peptides are merely working as coadjuvants and require the complementary action of other antifungal agents [78]. Encouragingly, the hRNase peptides were able to eradicate *C. albicans* biofilms at a low micromolar concentration (Figure 4). Moreover, we also found out that hRNase derived peptides are able to modify the expression of genes involved in hypha growth, biofilm formation and extracellular matrix synthesis. In addition, confocal microscopy analysis revealed that the treatment with the peptides RN3 and RN7 drastically reduces the biofilm viability to about 20%, altering the morphology of the mature biofilm structure (Figure 5). The principal virulence of *C. albicans* relies on its ability to alternate between yeast and hyphal form and generate a biofilm structure. As described above, the RN3 and RN7 peptides are not only able to remove pre-formed biofilms, but also to alter the expression of genes related to the biofilm formation. Upon RN3 and RN7 peptides treatment we observed the down-regulation of key genes involved in the different stages of biofilm development (*ALS3*, *CYR1*, *EFG1*, *ADH5* and *ZAP1*) (Figure 7), with the exception of the *CSH1* gene, which was upregulated after treatment with RN7. The up-regulation of *CSH1* expression was also reported in *C. albicans* biofilms exposed to peptide KP [75]. These changes in the expression level of genes involved in biofilm formation corroborate that hRNase derived peptides act in multiples stages to prevent biofilm formation. The suppression of the expression levels of some genes related to cAMP-PKA kinase pathways implicated in hyphal formation-, such as *CYR1* and *EFG1,* might indicate a potential target of interest of hRNase derived peptides; however, further studies will be necessary to fully understand their mechanism of action. 

On the other hand, removal of biofilms in vivo, is reported for salivary agglutinin and histatin and can be facilitated by the peptide binding to the cell polysaccharide external layer and cell agglutination [79]. Results with salivary histatins in animal models are promising and the antifungal peptide is proposed for topical therapy for oral candidiasis [79]. A shorter version of histatin 5 is currently in clinical trials. The mannose binding protein is a lectin also displaying a high affinity for candida wall that can induce the yeast cells agglutination with proved efficacy in a murine model [80,81,82]. No other examples of antimicrobial peptides endowed with an agglutinating activity for *Candida* cells are found in the literature. Therefore, our present results highlight the multiples targets of action of hRNase-derived peptides toward *C. albicans* cells and their potentiality. In particular, the RN3 peptide combines high agglutination and antibiofilm activities. Novel peptide analogues have been recently designed to enhance the peptide stability and resistance to proteases in vivo. Further work is currently ongoing to ensure the peptide druggability before considering any potential therapeutic application. 

## 4. Materials and Methods 

### 4.1. Peptide Synthesis

Peptides were synthesized as previously described [23]. Fmoc-protected amino acids and hexafluorophosphate benzotriazole tetramethyl uronium (HBTU) were obtained from Iris Biotech. Fmoc-Rink-amide (MBHA) resin was from Novabiochem. HPLC-grade acetonitrile (ACN) and peptide-synthesis-grade dimethylformamide (DMF), N,Ndi-isopropylethylamine (DIEA) and trifluoroacetic acid (TFA) were from Carlo Erba-SDS. Solid-phase peptide synthesis was done by Fmoc-based chemistry on Fmoc-Rink-amide (MBHA) resin (0.1 mmol) in a model 433 synthesizer (Applied Biosystems) running FastMoc protocols. Couplings used an 8-fold molar excess each of Fmoc-amino acid and HBTU and a 16-fold molar excess of DIEA. Side chains of trifunctional residues were protected with t-butyl (aspartate, glutamate, serine, threonine and tyrosine), t-butyloxycarbonyl (lysine and tryptophan), 2,2,4,6,7-pentamethyldihydrobenzofuran-5-sulfonyl (arginine) and trityl (asparagine, glutamine and histidine) groups. After chain assembly, full deprotection and cleavage were carried out with TFA/water/tri-isopropylsilane (95:2.5:2.5, by vol.) for 90 min at room temperature (25 °C). Peptides were isolated by precipitation with ice-cold diethyl ether and separated by centrifugation (3000× *g* for 20 min at 4 °C), dissolved in 0.1 M acetic acid, and freeze-dried. Analytical reversed-phase HPLC was performed on a Luna C18 column (4.6 mm × 50 mm, 3 μm; Phenomenex). Linear 5–60% gradients of solvent B (0.036% TFA in ACN) into solvent A (0.045% TFA in water) were used for elution at a flow rate of 1 mL/min and with UV detection at 220 nm. Preparative HPLC runs were performed on a Luna C18 column (21.2 mm × 250 mm, 10 μm; Phenomenex), using linear gradients of solvent B (0.1% in ACN) into solvent A (0.1%TFA in water), as required, with a flow rate of 25 mL/min. MALDI–TOF mass spectra were recorded in the reflector or linear mode in a Voyager DE-STR workstation (Applied biosystems) using an α-hydroxycinnamic acid matrix. Fractions of adequate (>90%) HPLC homogeneity and with the expected mass (supplementary Figures S5 and S6) were pooled and freeze-dried for subsequent experiments.

### 4.2. C. albicans Growth Conditions

*C. albicans* (ATCC 90028) cells were stored at −80 °C (15% glycerol). After thawing, cells were incubated overnight with agitation at 30 °C in Sabouraud Dextrose broth (Sigma S3306). Before each assay, cells were sub-cultured for 2–3 h to yield a mid-logarithmic phase culture. For the biofilms formation assay *C. albicans* cells were cultured in RPMI-1640 medium.

### 4.3. Minimum Fungicidal Concentration

*C. albicans* was cultured overnight in Sabouraud Dextrose broth at 30 °C, sub-cultured the next day in fresh media and grown to an optical density of 0.4 at 592 nm (mid log-phase). Cells were washed twice and diluted to ~2 × 10^5^ cells/mL. Peptides serially diluted were added to the cells from 20 to 0.1 µM final concentration. *C. albicans* was incubated at 30 °C during 4h in Sabouraud nutrient broth or PBS. Following, the samples were plated onto Sabouraud Petri dishes and incubated at 30 °C overnight. Antifungal activity was expressed as the MFC, defined as the lowest peptide concentration required for more than 99% of microorganism killing. MFC of each peptide was determined from two independent experiments performed in triplicate for each concentration.

### 4.4. Minimal Agglutination Activity (MAC)

*C. albicans* cells were grown at 30 °C to an OD_592_ of 1.0, diluted 10 times, centrifuged at 5000× *g* for 2 min, and resuspended in 1× PBS. An aliquot of 100 μL of the cellular suspension was treated with increasing peptide concentrations (from 0.01 to 20 μM) and incubated at 30 °C for 1 h. The aggregation behavior was observed by visual inspection, and the agglutinating activity is expressed as the minimum agglutinating concentration of the sample tested, as previously described [58]. Images were taken using a 50× stereoscope microscope.

### 4.5. Cell Viability Assay

Cell viability of *C. albicans* was assayed using the BacTiter-Glo^™^ Microbial Cell Viability kit (Promega), which measures the number of viable cells, by ATP quantification. ATP, as an indicator of metabolically active cells, is indirectly measured by a coupled luminescence detection assay. The luminescent signal is proportional to the amount of ATP required for the conversion of luciferin into oxyluciferin in the presence of luciferase. An overnight culture of *C. albicans* was used to inoculate fresh Sabouraud liquid culture, and logarithmic phase culture was grown to an OD_592_ of 0.2. RNase derived N-terminal peptides were added to 0.1 mL of cell culture at a final concentration from 0.025 to 20 μM. The *C. albicans* viability was followed after 4 h of incubation at 30 °C. 50 µL of incubation culture were mixed with 50 µL of BacTiter-Glo^TM^ reagent in a microtiter plate following the manufacturer instructions and incubated at room temperature for 10 min. Luminiscence was read on a Victor3 plate reader (PerkinElmer, Waltham, MA, USA) with a 1-s integration time. IC_50_ values were calculated by fitting the data to a dose-response curve.

### 4.6. Cell Survival Assay

*C. albicans* viability assay was performed using the Live⁄Dead^®^ microbial viability kit as previously described [32]. *Candida* strain was grown at 30 °C to ~5 × 10^6^ cells/mL, centrifuged at 5000× *g* for 5 min and resuspended in a 0.85% NaCl solution, in accordance with the manufacturer instructions. *C. albicans* cell culture was stained using a SYTO^®^9/propidium iodide 1:1 mixture. SYTO9/propidium iodide staining allowed us to monitor both the candidal viability in planktonic cell cultures and within the biofilm, as the SYTO9 dye is able to permeate the cell membrane and stain the nucleic acid of viable cells; whereas, when the cell membrane is damaged, propidium iodide can diffuse into the cell and displace SYTO9. The method allows the labelling of intact viable cells and membrane compromised cells, which are labelled in green and red respectively, referred to as live and dead cells. The viability kinetics were monitored using a Cary Eclipse Spectrofluorimeter (Varian Inc., Palo Alto, CA, USA). Cell viability profiles were registered after adding from 1 to 5 µM of final peptide concentration. To calculate the cell viability, the fluorescence in the range of 510–540 nm and 620–650 nm were integrated to obtain the SYTO^®^9 (live cell) and the propidium iodide (dead cell) signals respectively. Then, the percentage of live bacteria was represented as a function of time.

### 4.7. Cell Membrane Depolarization Assay

Membrane depolarization was assayed by monitoring the DiSC3 fluorescence intensity change in response to changes in transmembrane potential as described previously [33]. *C. albicans* cells were grown at 30 °C to the mid-exponential (OD_592_~0.4) and resuspended in 5 mM Hepes-KOH, 20 mM glucose and 100 mM KCl at pH 7.2. DiSC3 was added to a final concentration of 0.4 µM. Changes in the fluorescence for alteration of the cell plasma membrane potential were continuously monitored at 20 °C at an excitation wavelength of 620 nm and an emission wavelength of 670 nm. When the dye uptake was maximal, as indicated by a stable reduction in the fluorescence as a result of quenching of the accumulated dye in the membrane interior, protein in 5 mM Hepes-KOH buffer at pH 7.2 was added at a final peptide concentration from 1 to 5 μM and incubated for 50 min, to calculate the ED_50_ concentration. Maximum depolarization was calculated when the fluorescence signal was fully stabilized, and the depolarization percentage was calculated taking Triton X-100 at 10% as a maximum reference value. The time required to reach a stabilized maximum fluorescence reading was recorded for each condition, and the time required to achieve half of total membrane depolarization was estimated from the nonlinear regression curve. All conditions were assayed in duplicate.

### 4.8. Cell Membrane Permeabilization Activity

Membrane permeabilization was evaluated by using the Sytox^®^ Green uptake assay. Sytox® Green is a cationic cyanine dye which is unable to cross the membrane. When cells’ plasma membrane integrity is compromised, the dye enters into the cell, binding to DNA and triggering a large increase of the fluorescence signal. For Sytox^®^ Green assays, *C. albicans* cells were grown to mid-exponential growth phase at 30 °C and then centrifuged, washed, and resuspended in PBS. Cell suspensions in PBS (~2 × 10^6^ cells/mL) were incubated with 1 μM Sytox^®^ Green for 15 min in the dark prior to the influx assay. At 2 to 4 min after initiating data collection, proteins at 1 and 5 μM final concentration were added to the cell suspension, and the increase in Sytox^®^ Green fluorescence was measured (excitation wavelength at 485 nm and emission at 520 nm) for 50 min in a Cary Eclipse spectrofluorimeter. Fungal cell lysis with 10% Triton X-100 was taken as the maximum fluorescence reference value.

### 4.9. Cell Binding Assay

*C. albicans* was cultured overnight in Sabouraud Dextrose broth at 30 °C, sub-cultured the next day and grown to an optical density of 0.4 at 592 nm (mid log-phase). Cells were washed twice in PBS and adjusted to 2 × 10^6^ cells/mL. Cells and yeast β-d-Glucan (0.4 mg/mL) (Sigma) were respectively incubated in 100 μL of PBS at 30 °C with proteins and peptides at 1 μM final concentration during different periods of time up to 1 h. Following, the samples were centrifuged at 12,000× *g*. The supernatant samples were concentrated, and the presence of the proteins and peptides was checked by 15–18% SDS-PAGE and Coomassie Blue staining. Reference protein controls were treated following the same protocol in the absence of cells.

### 4.10. DNA Binding Assay

Gel retardation experiments were performed following an adaptation of a previously described protocol [83,84]. Briefly, aliquots of 300 ng of the plasmidic DNA (pET 28a) and increasing concentrations of peptides were mixed in 10 μL of binding buffer consisting of 5% glycerol, 10 mM Tris HCl (pH 8.0), 1 mM EDTA, 20 mM KCl, and 50 mg/mL BSA and incubated at room temperature for 20 min. Aliquots of each reaction were separated by electrophoresis on 1% agarose gels followed by ethidium bromide staining. The peptide-to-DNA weight ratios were 0:1, 2.5:1, 5:1, 10:1, 20:1 and 50:1 respectively. Gel retardation was visualized under UV illumination and recorded using gel Molecular Imager Gel Doc XRS.

### 4.11. Gene Expression Analysis

*C. albicans* gene expression of proteins implicated in common metabolic pathways was evaluated following incubation with 0.5 μM concentrations of RN3 and RN7 at 30 °C. The protein expression levels of *18s* ribosomal RNA, Glyceraldehyde 3-phospate dehydrogenase (*GAPDH*) and *KRE6* enzyme were examined. *KRE6* enzyme participates in the synthesis of β-1,6-glucan ramifications to the β-1,3-glucan chain and provides a tight mesh structure to the cell wall. Total RNA was extracted using a *mirVana*™ Isolation Kit. *C. albicans* cell cultures (3 mL) at log-phase (OD_592_ of 0.2) were incubated with the peptides during 2 h. After incubation, cells were sedimented and resuspended in lysis buffer, 10% SDS and Phenol:Chloroform: isoamyl alcohol (IAA) and mixed with zirconia beads. RNA isolation was done according to manufacturer’s instructions. The amount of RNA extracted was quantified using a NanoDrop 2000 spectrophotometer (Thermo Scientific, USA). A yield of about 500 ng/μL was obtained per culture sample. Complementary DNA (cDNA) synthesis was the first step of the two-step quantitative reverse transcriptase Polymerase Chain Reaction (RT-qPCR). cDNA was generated by the enzyme reverse transcriptase (RT). 1000 ng of total RNA was used to synthesize cDNA according to the manufacture instruction of iScript™ cDNA Synthesis Kit. Used primers are listed in supplementary Table S3.

Additionally, we investigated the peptide-induced changes in transcription level of genes related to biofilm formation. Expression of hyphal-specific and biofilm-associated genes: agglutinin-like protein precursor 3 (*ALS3*) and their transcriptional regulators (*CYR1* and *EFG1*) was analyzed. In addition, genes related to the production of extracellular matrix (*ADH5, CSH1, GSC1* and *ZAP1*) were evaluated. *C. albicans* cells (OD_592_ ~0.4) were grown in the absence or presence of 10 μM of each peptide in 6-well plates for 24 h at 37 °C. Next, wells were washed with PBS and adhered cells were removed from the bottom of the wells by scraping. Total RNA was extracted from sessile cells, as described above, and stored at −80 °C. The quality and quantity of the extracted RNA were determined spectrophotometrically. The cDNA library from the total RNA extract (100 ng) was build using the Reverse Transcription System kit (Promega), following manufacturer’s instructions.

Gene expression level was quantified by Real Time Quantitative Polymerase Chain Reaction (RT-qPCR). For each reaction, 1μL of cDNA (10 to 50 ng) was mixed with 2 μL of free RNase water, 5 μL of iTaq Universal Master SYBR Green Supermix and 1 μL of each primer of interest (500 nM) in a total volume of 10 μL. Reactions were incubated at 60 °C for 1 min and 95 °C for 10 min, followed by 40 cycles of 95 °C for 15 s and 60 °C for 1 min. All the primers used for RT-qPCR were from Bio-Rad. *ACTIN* was used as a housekeeping gene for data normalization. All the primers used for RT-qPCR were from Bio-Rad. Data analysis was performed using Bio-Rad CFX Manager. Three independent experiments were performed, with their own technical triplicate. The figures of gene level expression were designed by *Graphpad Prism6* software.

### 4.12. Biofilm Growth

Biofilms of *C. albicans* were prepared on pre-sterilized, polystyrene, flat bottomed 96-well microtiter plates following a modification of a previously described protocol [85,86]. First, a standardized yeast suspension (10^7^ cells/mL) of *C. albicans* was prepared by suspending colonies from 12 h-old culture in RPMI-1640 medium and adjusted to an optical density of 0.4 at 520 nm. 100 μL of yeast suspension was dispensed into each well of microtiter plate using a multichannel pipette and the plates were incubated at 37 °C for 90 min to allow adherence of yeast on the surface of each well. After the adhesion phase, the non-adherent cells were removed, and each well was washed twice with 150 μL PBS. An aliquot of 100 μL of RPMI 1640 medium was transferred to each washed well and the plates were incubated at 37 °C for 24 h to allow biofilm formation. 

### 4.13. Biofilm Viability Test

Following the biofilm formation phase, the medium was aspirated, and each well was gently washed twice with 200 μL of PBS to remove non-adherent cells. 200 μL of each peptide with concentrations ranging from 5, 10 and 20 times of the determined MFC were added to the respective wells and the plates were incubated as described above for 24 h. Colistin and amphotericin B were assayed as positive controls of inhibition. Wells with biofilm and media alone and biofilm-free wells were included as positive and background references. After treatment with the peptides, the medium was removed, and each well was washed twice with 200 μL PBS and 90 µL of RPMI-1640 medium were added. Then, 10 μL of *PrestoBlue*^TM^ was added to prewashed biofilm and control wells and incubated for 10 min in the dark. Changes in the fluorescence signal were quantified at excitation/emission of 535–615 nm using a 96-well plate reader (Victor^3^, Perkin-Elmer, Waltham, MA, USA).

### 4.14. Biofilm Mass Eradication

Biofilm mass eradication was quantified by the crystal violet staining assay according to [85,87], with slight modifications. Briefly, the biofilm- coated wells of microtiter plates were washed twice with 200 μL of PBS and then air-dried for 45 min. Then, each of the washed wells was stained with 100 μL of 0.4% aqueous crystal violet solution for 45 min. Afterwards, each well was washed four times with 350 μL of distilled water and immediately destained with 200 μL of 96% ethanol. After 45 min of destaining, 100 μL of destaining solution was transferred to a new well and the amount of the crystal violet was measured with a microtiter plate reader Victor3 at 570 nm. 

### 4.15. Confocal Microscopy

Biofilms were visualized by confocal microscopy following *Live/Dead* staining (Molecular Probes, Eugene, OR, USA). Overnight culture of *C. albicans* cells were centrifuged at 5000× *g* for 5 min, washed, resuspended in fresh RPMI-1640 medium and adjusted to an OD_592_ of 0.4. The candidal suspension was added to a plate coverslip system 35-cm^2^ and incubated at 37 °C for 24 h. At 24 h, *C. albicans* cells were washed three times with PBS and incubated during 4h with 20 μM of each peptide resuspended in HEPES 10 mM pH 7.5 at 37 °C. After incubation, the wells were washed three times with PBS to remove planktonic *C. albicans* cells. Then, the biofilms were prestained using the SYTO9/propidium iodide 1:1 mixture provided by the *Live/Dead* staining kit and incubated in the dark at 37 °C for 15 min. Changes were imaged using a laser scanning confocal microscope (Olympus FluoView 1000 equipped with a UPlansApo 60× lens in 1.4 oil immersion objective, UK). Mortality was calculated as the mean area of 10 randomly selected fields and quantified using ImageJ software (Oxford Instruments, Zurich, Switzerland). Unspecific background was subtracted, and particles with a diameter larger than 4 μm were analyzed. Biofilm three-dimensional reconstruction and estimation of the biofilm depth were performed with the IMARIS Bitplane imaging software. 

### 4.16. Cytotoxicity Assay

The peptide cytoxicity was evaluated using the human monocytic THP1 cell line. The assay was performed in 96-well cell culture flat-bottom plates (Costar; Appleton Woods) in triplicate. THP-1 cultures were induced to macrophages by adding 2 ng/mL of colony-stimulating factor (R&D Systems, Germany). Peptides were serially diluted from 50 to 0.1 µM and added to 5 × 10^5^ cells/mL. After 48 h of incubation, the cells were washed twice with 1× PBS, and fresh RPMI-1640 complete medium was added. Plates were then treated with 30 μL of a freshly prepared 0.01% resazurin solution and incubated overnight at 37 °C. The following day the change in color was observed and the fluorescence intensity was measured (λ_ex_560 nm, λ_em_590 nm). The 50% growth inhibitory concentration (GIC_50_) was determined based on the resazurin fluorescence signal.

### 4.17. Statistical Analysis

Statistical analysis was performed using Prism6^®^ by one-way ANOVA test followed by Dunnett’s Multiple Comparison Test. The results were from three independent experiments. A *p* value < 0.05 and *p* < 0.001 were considered statistically significant. 

## 5. Conclusions

We can conclude that the pleiotropic antimicrobial mechanism of action of the hRNase-derived peptides offers multiple advantages to be considered. Results highlight RN3 as a promising lead peptide for the development of novel antifungal agents. In particular, the peptide combines potent killing activity on planktonic *C. albicans* by cell binding and membrane permeabilization, together with the ability to agglutinate cells, inhibit biofilm formation and eradicate the mature stablished communities at low micromolar concentrations. Therefore, hRNases derived peptides are proposed as suitable candidates to develop novel and safe antifungal agents.

## Figures and Tables

**Table 1:** Physicochemical and biological properties of the hRNase-derived peptides.

Peptide ^†^	Sequence	Hydrophobicity	pI	K/R	Protein Antimicrobial Activity *	Cytotoxicity (THP1) # GIC_50_ (μM)
RN1	---KESRAKKFQRQHMDSDSSPSSSSTYSNQMMRRRNMTQGRSKPVNTFVH	−1.575	11.40	4/6	n.r	>50
RN2	KPPQFTWAQWFETQHINMTSQ------QSTNAMQVINNYQRRSKNQNTFLL	−1.069	10.28	2/2	-	>50
RN3	RPPQFTRAQWFAIQHISLNPP------RSTIAMRAINNYRWRSKNQNTFLR	−0.764	11.88	1/7	+++ ^Ŧ^	>50
RN3K	KPPQFTKAQWFAIQHISLNPP------KSTIAMKAINNYKWKSKNQNTFLK	−0.824	11.02	8/-	+++	>50
RN4	--QDGMY-QRFLRQHVHPEET-GGSDRYSNLMMQRRKMTLYHSKRFNTFIH	−1.228	10.15	2/6	n.r	>50
RN5	--QDNSRYTHFLTQHYDAKPQ-GRDDRYSESIMRRRGLTS-PSKDINTFIH	−1.430	9.40	2/6	-	>50
RN6	WPKRLTKAHWFEIQHIQPSPL------QSNRAMSGINNYTQHSKHQNTFLH	−1.096	10.45	3/2	+++	>50
RN7	KPKGMTSSQWFKIQHMQPSPQ------ASNSAMKNINKHTKRSKDLNTFLH	−1.209	10.75	6/1	+++ ^Ŧ^	>50
RN7R	RPRGMTSSQWFRIQHMQPSPQ------ASNSAMRNINRHTRRSRDLNTFLH	−1.302	12.88	-/8	+++	>50
RN8	KPKDMTSSQWFKTQHVQPSPQ------ASNSAMSIINKYTERSKDLNTFLH	−1.044	9.70	5/1	+	>50

† The N-terminal derived peptides are numbered according to their respective parental proteins, K and R indicate Lys and Arg peptide enrichment. * Antimicrobial activity of the parental proteins tested against bacterial strains (*E. coli* and *S. aureus*), obtained from [23,30]. Antibacterial activity range: + MIC_100_ (μM) > 10, +++ MIC_100_ (μM) 0.3- <10. Ŧ Positive antifungal activity of parental proteins against *C. albicans*, as previously determined [21]; n.r: Not reported. # The peptide cytotoxicity was evaluated by their 50% Growth Inhibition Concentration (GIC_50_) on THP1 cells.

**Table 2:** Antifungal Activities of hRNases Peptides on *C. albicans.*

hRNase Peptide	MFC_100_ (μM)		IC_50_ (µM)
	Sabouraud Broth	PBS	
RN1	3.75 ± 0.50	3.75 ± 0.30	2.10 ± 0.20
RN2	>20	>20	>20
RN3	0.62 ± 0.20	0.62 ± 0.10	0.22 ± 0.05
RN4	4.00 ± 0.50	3.75 ± 0.20	1.80 ± 0.30
RN5	>20	>20	>20
RN6	2.00 ± 0.50	1.75 ± 0.30	1.80 ± 0.20
RN7	0.93 ± 0.20	0.93 ± 0.10	0.45 ± 0.10
RN8	>10	>10	>10

Minimal fungicidal concentration (MFC_100_) was determined as the lowest concentration of peptide that killed at least 99.9% of the initial inoculum. Values were calculated by Colony Forming Unit (CFU) counting on plated Petri dishes as described in the methodology. *C. albicans* cultures were treated with the proteins diluted in either the Sabouraud nutrient growth media or in a phosphate saline buffer (PBS). Values for 50% Inhibition Concentration (IC_50_) for planktonic fungal cells were determined using the Bactiter-Glo™ kit as detailed in the Materials and Methods section. Values are given as mean ± SEM.

**Table 3:** Cell Membrane Depolarization, Permeabilization and Antimicrobial Activities of hRNases Peptides on *C. albicans*.

hRNase Peptide	Membrane Depolarization		Membrane Permeabilization		Cell Survival
	ED_50_ (µM)	Depol_max_ *	Max. Perm. (AU) ^1^	Perm. (%) ^2^	% ^¥^
RN1	4.63 ± 0.70	65.2 ± 4.6	26.1 ± 0.4	8.3 ± 0.4	67 ± 1
RN2	3.63 ± 1.20	28.6 ± 5.7	45.2 ± 0.8	12.4 ± 0.2	72 ± 0.5
RN3	0.60 ± 0.40	100 ± 3.2	166 ± 2	73.2 ± 1	15 ± 2
RN4	3.58 ± 1.60	76.2 ± 4.8	117 ± 2	38.7 ± 0.1	65 ± 10
RN5	1.51 ± 0.10	39.1 ± 5.1	66.5 ± 0.6	21.8 ± 0.4	39 ± 0.5
RN6	0.62 ± 0.10	86.2 ± 5.2	135 ± 1	48.4 ± 0.8	26 ± 2
RN7	0.53 ± 0.18	71.5 ± 3.7	136 ± 0.4	53.9 ± 0.5	12 ± 1
RN8	0.15 ± 0.20	36.1 ± 4.4	33.4 ± 0.4	11.2 ± 0.2	28 ± 1

ED_50_ depolarization values were calculated with peptide concentrations from 0.1 to 5 µM. * Maximum fluorescence value reached at the final incubation time with 2 µM of the peptides. Membrane depolarization and permeabilization were performed using the membrane potential-sensitive DiSC3 fluorescent probe and *Sytox^®^ Green,* respectively, as described in the Materials and Methods section. Values are given as mean ± SEM. ^¥^ Survival percentage at final incubation time (120 min) was evaluated using the Live/Dead^R^ kit. ^1^ Arbitrary fluorescence unit (AU) values are indicated for maximum membrane depolarization and permeabilization. ^2^ The calculated percentages refer to the maximum values achieved at final incubation time (50 min), referred to the positive control (10 % of Triton X-100). All values are averaged from three replicates of two independent experiments.

## Supplementary Materials

Supplementary materials can be found at https://www.mdpi.com/1422-0067/20/18/4558/s1.

## Abbreviations

CFU	Colony forming units
EDTA	Ethylenediaminetetraacetic acid
SDS-PAGE	Sodium dodecyl sulfate polyacrylamide gel electrophoresis
ATP	Adenosine triphosphate
DiSC3	3,3′-Dipropylthiadicarbocyanine Iodide
IC	Inhibition Concentration
MFC	Minimum Fungicidal Concentration
MAC	Minimum Agglutination Concentration
PI	Propidium iodide

## References

[1] Jabra-Rizk M.A., Kong E.F., Tsui C., Nguyen M.H., Clancy C.J., Fidel P.L., Noverr M., Noverr M. (2016). Candida albicans Pathogenesis: Fitting within the Host-Microbe Damage Response Framework. Infect. Immun..

[2] Gulati M., Nobile C.J. (2016). Candida albicans biofilms: development, regulation, and molecular mechanisms. Microbes Infect..

[3] Costa-Orlandi C., Sardi J., Pitangui N., de Oliveira H., Scorzoni L., Galeane M., Medina-Alarcón K., Melo W., Marcelino M., Braz J. (2017). Fungal Biofilms and Polymicrobial Diseases. J. Fungi.

[4] Kim D.J., Lee M.W., Choi J.S., Lee S.G., Park J.Y., Kim S.W. (2017). Inhibitory activity of hinokitiol against biofilm formation in fluconazole-resistant Candida species. PLoS ONE.

[5] Nobile C.J., Johnsonb A.D. (2015). Candida albicans biofilms and human disease. Annu. Rev. Microbiol..

[6] Zasloff M. (2002). Antimicrobial peptides of multicellular organisms. Nature.

[7] Zasloff M. (2002). Antimicrobial peptides in health and disease. N. Engl. J. Med..

[8] Gordon Y.J., Romanowski E.G., McDermott A.M. (2005). A review of antimicrobial peptides and their therapeutic potential as anti-infective drugs. Curr. Eye Res..

[9] Wang G., Li X., Wang Z. (2009). APD2: The updated antimicrobial peptide database and its application in peptide design. Nucleic Acids Res..

[10] Boman H.G. (2003). Antibacterial peptides: Basic facts and emerging concepts. J. Intern. Med..

[11] Fosgerau K., Hoffmann T. (2015). Peptide therapeutics: Current status and future directions. Drug Discov. Today.

[12] Sierra J.M., Fusté E., Rabanal F., Vinuesa T., Viñas M. (2017). An overview of antimicrobial peptides and the latest advances in their development. Expert Opin. Biol. Ther..

[13] Mahlapuu M., Håkansson J., Ringstad L., Björn C. (2016). Antimicrobial Peptides: An Emerging Category of Therapeutic Agents. Front. Cell. Infect. Microbiol..

[14] Batoni G., Maisetta G., Lisa Brancatisano F., Esin S., Campa M. (2011). Use of Antimicrobial Peptides Against Microbial Biofilms: Advantages and Limits. Curr. Med. Chem..

[15] Lázár V., Martins A., Spohn R., Daruka L., Grézal G., Fekete G., Számel M., Jangir P.K., Kintses B., Csörgő B. (2018). Antibiotic-resistant bacteria show widespread collateral sensitivity to antimicrobial peptides. Nat. Microbiol..

[16] Bondaryk M., Staniszewska M., Zielińska P., Urbańczyk-Lipkowska Z. (2017). Natural Antimicrobial Peptides as Inspiration for Design of a New Generation Antifungal Compounds. J. Fungi.

[17] Swidergall M., Ernst J.F. (2014). Interplay between Candida albicans and the antimicrobial peptide armory. Eukaryot. Cell.

[18] Boix E., Nogués M.V. (2007). Mammalian antimicrobial proteins and peptides: overview on the RNase A superfamily members involved in innate host defence. Mol. Biosyst..

[19] Pulido D., Prats-Ejarque G., Villalba C., Albacar M., González-López J.J., Torrent M., Moussaoui M., Boix E. (2016). A novel RNase 3/ECP peptide for Pseudomonas aeruginosa biofilm eradication that combines antimicrobial, lipopolysaccharide binding, and cell-agglutinating activities. Antimicrob. Agents Chemother..

[20] Pulido D., Prats-Ejarque G., Villalba C., Albacar M., Moussaoui M., Andreu D., Volkmer R., Torrent M., Boix E. (2018). Positional scanning library applied to the human eosinophil cationic protein/RNase3 N-terminus reveals novel and potent anti-biofilm peptides. Eur. J. Med. Chem..

[21] Salazar V.A., Arranz-Trullén J., Navarro S., Blanco J.A., Sánchez D., Moussaoui M., Boix E. (2016). Exploring the mechanisms of action of human secretory RNase 3 and RNase 7 against Candida albicans. Microbiologyopen.

[22] Lu L., Li J., Moussaoui M., Boix E. (2018). Immune modulation by human secreted RNases at the extracellular space. Front. Immunol..

[23] Torrent M., Pulido D., Valle J., Nogués M.V.V., Andreu D., Boix E. (2013). Ribonucleases as a host-defence family: Evidence of evolutionarily conserved antimicrobial activity at the N-terminus. Biochem. J..

[24] Torrent M., de la Torre B.G., Nogués V.M., Andreu D., Boix E. (2009). Bactericidal and membrane disruption activities of the eosinophil cationic protein are largely retained in an N-terminal fragment. Biochem. J..

[25] Torrent M., Pulido D., de la Torre B.G., Garcia-Mayoral M.F., Nogues M.V., Bruix M., Andreu D., Boix E. (2011). Refining the eosinophil cationic protein antibacterial pharmacophore by rational structure minimization. J. Med. Chem..

[26] Schmidt N., Mishra A., Lai G.H., Wong G.C.L. (2010). Arginine-rich cell-penetrating peptides. FEBS Lett..

[27] Kerkis A., Hayashi M.A.F., Yamane T., Kerkis I. (2006). Properties of cell penetrating peptides (CPPs). IUBMB Life.

[28] Wang G., Li X., Wang Z. (2016). APD3: the antimicrobial peptide database as a tool for research and education. Nucleic Acids Res..

[29] Yin L.M., Edwards M.A., Li J., Yip C.M., Deber C.M. (2012). Roles of hydrophobicity and charge distribution of cationic antimicrobial peptides in peptide-membrane interactions. J. Biol. Chem..

[30] Boix E., Salazar V.A., Torrent M., Pulido D., Nogués M.V., Moussaoui M. (2012). Structural determinants of the eosinophil cationic protein antimicrobial activity. Biol. Chem..

[31] Torrent M., Sánchez D., Buzón V., Nogués M.V., Cladera J., Boix E. (2009). Comparison of the membrane interaction mechanism of two antimicrobial RNases: RNase 3/ECP and RNase 7. Biochim. Biophys. Acta Biomembr..

[32] Torrent M., Badia M., Moussaoui M., Sanchez D., Nogués M.V., Boix E. (2010). Comparison of human RNase 3 and RNase 7 bactericidal action at the Gram-negative and Gram-positive bacterial cell wall. FEBS J..

[33] Torrent M., Navarro S., Moussaoui M., Nogues M.V., Boix E. (2008). Eosinophil cationic protein high-affinity binding to bacteria-wall lipopolysaccharides and peptidoglycans. Biochemistry.

[34] García-Mayoral M.F., Moussaoui M., de la Torre B.G., Andreu D., Boix E., Nogués M.V., Rico M., Laurents D.V., Bruix M. (2010). NMR structural determinants of eosinophil cationic protein binding to membrane and heparin mimetics. Biophys. J..

[35] García-Mayoral M.F., Canales Á., Díaz D., López-Prados J., Moussaoui M., De Paz J.L., Angulo J., Nieto P.M., Jiménez-Barbero J., Boix E. (2013). Insights into the glycosaminoglycan-mediated cytotoxic mechanism of eosinophil cationic protein revealed by NMR. ACS Chem. Biol..

[36] Torrent M., Nogués M.V., Boix E. (2011). Eosinophil cationic protein (ECP) can bind heparin and other glycosaminoglycans through its RNase active site. J. Mol. Recognit..

[37] Avci F.G. (2018). Membrane Active Peptides and Their Biophysical Characterization. Biomolecules.

[38] Torrent M., Cuyás E., Carreras E., Navarro S., López O., De La Maza A., Nogués M.V., Reshetnyak Y.K., Boix E. (2007). Topography studies on the membrane interaction mechanism of the eosinophil cationic protein. Biochemistry.

[39] Gow N.A.R., Van De Veerdonk F.L., Brown A.J.P., Netea M.G., Van De Veerdonk F.L., Brown A.J.P., Netea M.G. (2013). Candida albicans morphogenesis and host defence:discriminating invasion from colonization. Nat. Rev. Microbiol..

[40] Pierce C.G., Vila T., Romo J.A., Montelongo-jauregui D., Wall G., Ramasubramanian A., Lopez-ribot J.L. (2017). The Candida albicans Biofilm Matrix: Composition, Structure and Function. J. Fungi.

[41] Borghi E., Borgo F., Morace G. (2016). Fungal Biofilms: Update on Resistance. Advances in Experimental Medicine and Biology.

[42] Cavalheiro M., Teixeira M.C. (2018). Candida Biofilms: Threats, Challenges, and Promising Strategies. Front. Med..

[43] Lum K.Y., Tay S.T., Le C.F., Lee V.S., Sabri N.H., Velayuthan R.D., Hassan H., Sekaran S.D. (2015). Activity of novel synthetic peptides against Candida albicans. Sci. Rep..

[44] Teixeira-Santos R., Ricardo E., Branco R.J., Azevedo M.M., Rodrigues A.G., Pina-Vaz C. (2016). Unveiling the synergistic interaction between liposomal amphotericin B and colistin. Front. Microbiol..

[45] Maiolo E.M., Tafin U.F., Borens O., Trampuz A. (2014). Activities of fluconazole, caspofungin, anidulafungin, and amphotericin b on planktonic and biofilm candida species determined by microcalorimetry. Antimicrob. Agents Chemother..

[46] Hamill R.J. (2013). Amphotericin B formulations: A comparative review of efficacy and toxicity. Drugs.

[47] Hancock R.E.W., Sahl H.-G. (2006). Antimicrobial and host-defense peptides as new anti-infective therapeutic strategies. Nat. Biotechnol..

[48] Li J., Koh J.-J., Liu S., Lakshminarayanan R., Verma C.S., Beuerman R.W. (2017). Membrane Active Antimicrobial Peptides: Translating Mechanistic Insights to Design. Front. Neurosci..

[49] Ciociola T., Giovati L., Conti S., Santinoli C., Polonelli L. (2016). Natural and synthetic peptides with antifungal activity. Future Med. Chem..

[50] Nett J.E., Zarnowski R., Cabezas-Olcoz J., Brooks E.G., Bernhardt J., Marchillo K., Mosher D.F., Andes D.R. (2015). Host contributions to construction of three device-associated Candida albicans biofilms. Infect. Immun..

[51] Pulido D., Arranz-Trullén J., Prats-Ejarque G., Velázquez D., Torrent M., Moussaoui M., Boix E. (2016). Insights into the Antimicrobial Mechanism of Action of Human RNase6: Structural Determinants for Bacterial Cell Agglutination and Membrane Permeation. Int. J. Mol. Sci..

[52] Pulido D., Torrent M., Andreu D., Nogués M.V., Boix E., Nogues M.V., Boix E., Nogués M.V., Boix E. (2013). Two human host defense ribonucleases against mycobacteria, the eosinophil cationic protein (RNase 3) and RNase 7. Antimicrob. Agents Chemother..

[53] Rosenberg H.F. (1995). Recombinant human eosinophil cationic protein. Ribonuclease activity is not essential for cytotoxicity. J. Biol. Chem..

[54] Torrent M., Pulido D., Nogués M.V., Boix E. (2012). Exploring new biological functions of amyloids: bacteria cell agglutination mediated by host protein aggregation. PLoS Pathog..

[55] Pizzo E., D’Alessio G. (2007). The success of the RNase scaffold in the advance of biosciences and in evolution. Gene.

[56] Rosenberg H.F. (2008). RNase A ribonucleases and host defense: An evolving story. J. Leukoc. Biol..

[57] Becknell B., Eichler T.E., Beceiro S., Li B., Easterling R.S., Carpenter A.R., James C.L., McHugh K.M., Hains D.S., Partida-Sanchez S. (2014). Ribonucleases 6 and 7 have antimicrobial function in the human and murine urinary tract. Kidney Int..

[58] Pulido D., Moussaoui M., Andreu D., Nogués M.V., Torrent M., Boix E. (2012). Antimicrobial action and cell agglutination by the eosinophil cationic protein are modulated by the cell wall lipopolysaccharide structure. Antimicrob. Agents Chemother..

[59] Pulido D., Moussaoui M., Nogués M.V., Torrent M., Boix E. (2013). Towards the rational design of antimicrobial proteins: Single point mutations can switch on bactericidal and agglutinating activities on the RNase A superfamily lineage. FEBS J..

[60] Chao T.-Y., Raines R.T. (2011). Mechanism of ribonuclease A endocytosis: Analogies to cell-penetrating peptides. Biochemistry.

[61] Bahnsen J.S., Franzyk H., Sandberg-Schaal A., Nielsen H.M. (2013). Antimicrobial and cell-penetrating properties of penetratin analogs: Effect of sequence and secondary structure. Biochim. Biophys. Acta Biomembr..

[62] Zhu Y., Mohapatra S., Weisshaar J.C. (2018). Rigidification of the Escherichia coli cytoplasm by the human antimicrobial peptide LL-37 revealed by superresolution fluorescence microscopy. Proc. Natl. Acad. Sci. USA.

[63] Muñoz-Camargo C., Salazar V., Barrero L., Camargo S., Mosquera A., Mitrani E., Groot H., Boix E. (2018). Unveiling the multifaceted mechanisms of antibacterial activity of Buforin II and Frenatin 2.3S peptides from skin micro-organs of Sphaenorhynchus lacteus (Hylidae). Int. J. Mol. Sci..

[64] Li L., Sun J., Xia S., Tian X., Cheserek M.J., Le G. (2016). Mechanism of antifungal activity of antimicrobial peptide APP, a cell-penetrating peptide derivative, against Candida albicans: intracellular DNA binding and cell cycle arrest. Appl. Microbiol. Biotechnol..

[65] Tsai P.W., Cheng Y.L., Hsieh W.P., Lan C.Y. (2014). Responses of Candida albicans to the human antimicrobial peptide LL-37. J. Microbiol..

[66] Wang T., Xiu J., Zhang Y., Wu J., Ma X., Wang Y., Guo G., Shang X. (2017). Transcriptional responses of Candida albicans to antimicrobial peptide MAF-1A. Front. Microbiol..

[67] Theberge S., Semlali A., Alamri A., Leung K.P., Rouabhia M.C. (2013). albicans growth, transition, biofilm formation, and gene expression modulation by antimicrobial decapeptide KSL-W. BMC Microbiol..

[68] Morici P., Fais R., Rizzato C., Tavanti A., Lupetti A. (2016). Inhibition of Candida albicans biofilm formation by the synthetic lactoferricin derived peptide hLF1-11. PLoS ONE.

[69] Deveau A., Hogan D.A. (2011). Linking Quorum Sensing Regulation and Biofilm Formation by Candida albicans. Quorum Sensing.

[70] Kyriakidis I., Tragiannidis A., Munchen S., Groll A.H. (2017). Clinical hepatotoxicity associated with antifungal agents. Expert Opin. Drug Saf..

[71] Graham C.E., Cruz M.R., Garsin D.A., Lorenz M.C. (2017). Enterococcus faecalis bacteriocin EntV inhibits hyphal morphogenesis, biofilm formation, and virulence of Candida albicans. Proc. Natl. Acad. Sci. USA.

[72] Pletzer D., Coleman S.R., Hancock R.E.W. (2016). Anti-biofilm peptides as a new weapon in antimicrobial warfare. Curr. Opin. Microbiol..

[73] Ribeiro S.M., Felício M.R., Boas E.V., Gonçalves S., Costa F.F., Samy R.P., Santos N.C., Franco O.L. (2016). New frontiers for anti-biofilm drug development. Pharmacol. Ther..

[74] Mathur H., Field D., Rea M.C., Cotter P.D., Hill C., Ross R.P. (2018). Fighting biofilms with lantibiotics and other groups of bacteriocins. npj Biofilms Microbiomes.

[75] Paulone S., Ardizzoni A., Tavanti A., Piccinelli S., Rizzato C., Lupetti A., Colombari B., Pericolini E., Polonelli L., Magliani W. (2017). The synthetic killer peptide KP impairs Candida albicans biofilm in vitro. PLoS ONE.

[76] Helmerhorst E.J., Troxler R.F., Oppenheim F.G. (2001). The human salivary peptide histatin 5 exerts its antifungal activity through the formation of reactive oxygen species. Proc. Natl. Acad. Sci. USA.

[77] Lupetti A., Paulusma-Annema A., Senesi S., Campa M., Van Dissel J.T., Nibbering P.H. (2002). Internal thiols and reactive oxygen species in candidacidal activity exerted by an N-terminal peptide of human lactoferrin. Antimicrob. Agents Chemother..

[78] Hua J., Yamarthy R., Felsenstein S., Scott R.W., Markowitz K., Diamond G. (2010). Activity of antimicrobial peptide mimetics in the oral cavity: I. Activity against biofilms of Candida albicans. Mol. Oral Microbiol..

[79] Baker O.J., Edgerton M., Kramer J.M., Ruhl S. (2014). Saliva-microbe interactions and salivary gland dysfunction. Adv. Dent. Res..

[80] Ip W., Lau Y. (2004). Role of mannose-binding lectin in the innate defense against Candida albicans: Enhancement of complement activation, but lack of opsonic function, in phagocytosis by human dendritic cells. J. Infect. Dis..

[81] Clemons K.V., Martinez M., Axelsen M., Thiel S., Stevens D.A. (2011). Efficacy of recombinant human mannose binding lectin alone and in combination with itraconazole against murine Candida albicans vaginitis. Immunol. Investig..

[82] Hammad N.M., El Badawy N.E., Ghramh H.A., Al Kady L.M. (2018). Mannose-Binding Lectin: A Potential Therapeutic Candidate against Candida Infection. Biomed Res. Int..

[83] Park C.B., Kim H.S., Kim S.C. (1998). Mechanism of action of the antimicrobial peptide buforin II: buforin II kills microorganisms by penetrating the cell membrane and inhibiting cellular functions. Biochem. Biophys. Res. Commun..

[84] Jang S.A., Kim H., Lee J.Y., Shin J.R., Kim D.J., Cho J.H., Kim S.C. (2012). Mechanism of action and specificity of antimicrobial peptides designed based on buforin IIb. Peptides.

[85] Jin Y., Yip H.K., Samaranayake Y.H., Yau J.Y., Samaranayake L.P. (2003). Biofilm-forming ability of Candida albicans is unlikely to contribute to high levels of oral yeast carriage in cases of human immunodeficiency virus infection. J. Clin. Microbiol..

[86] Silva S., Henriques M., Oliveira R., Williams D., Azeredo J. (2010). In vitro biofilm activity of non-Candida albicans Candida species. Curr. Microbiol..

[87] Peeters E., Nelis H.J., Coenye T. (2008). Comparison of multiple methods for quantification of microbial biofilms grown in microtiter plates. J. Microbiol. Methods.

