# Supplementary material for: Insight into the Antifungal Mechanism of Action of Human RNase N-terminus Derived Peptides
Source: Int J Mol Sci. 2019 Sep 14;20(18):4558. doi: 10.3390/ijms20184558 (PMC6770517; doi:10.3390/ijms20184558)
Supplement: Supplementary file 1 [file ijms-20-04558-s001.pdf]

Insight into the antifungal mechanism of action of human RNase N-terminus derived peptides

Salazar, Vivian A[1,2*]; Arranz-Trullén, Javier[1]; Prats-Ejarque, Guillem[1]; Torrent, Marc[1]; Andreu David[3]; Pulido, David[1,4] and Boix, Ester[1*]

[1]*Department of Biochemistry and Molecular Biology, Faculty of Biosciences, Universitat Autònoma de Barcelona, 08193 Cerdanyola del Vallès, Spain*

[2] *Present address: Universidad de Los Andes, 111711 Bogotá, Colombia*

[3]*Department of Experimental and Health Sciences, Universitat Pompeu Fabra, Dr. Aiguader 88, 08003 Barcelona, Spain*

[4]*Present address: The Jenner Institute, University of Oxford, Old Road Campus Research Building, Oxford, OX3 7DQ UK. Oxford*

Supplementary figures and tables:

Supplementary Table S1 Antimicrobial activities of Lys and Arg peptide variants of RN3 and RN7

hRNase peptide	MFC_{100} (µM)		IC_{50} (µM)
	Sabouraud broth	PBS	
RN3K	4.25 ± 0.30	4.25 ± 0.30	2.08 ± 0.27
RN7R	2.83 ± 0.10	2.83 ± 0.10	1.62 ± 0.32

Minimal fungicidal concentration (MFC_{100}) was determined as the lowest concentration of peptide that killed at least 99.9% of the initial inoculum. Values were calculated by CFU counting on plated Petri dishes as described in the methodology. *C. albicans* cultures were treated with the proteins diluted in either the Sabouraud nutrient growth media or in a phosphate saline buffer (PBS). IC_{50} was determined using the Bactiter-Glo™ kit as detailed in the Materials and Methods section.

Supplementary Table S2. Minimal agglutination concentration (MAC) of hRNases' peptides.

**hRNase peptide**	**MAC (μM)**
**RN1**	1.80 ± 0.40
**RN2**	0.40 ± 0.10
**RN3**	< 0.1
**RN4**	0.40 ± 0.10
**RN5**	1.80 ± 0.40
**RN6**	0.40 ± 0.10
**RN7**	0.90 ± 0.30
**RN8**	0.90 ± 0.20

Minimal agglutination concentration (MAC) was calculated as the lowest peptide concentration where *C. albicans* cells aggregation is visible to the naked eye. Values are averaged from three replicates of two independent experiments. Values are given as mean ± SEM.

Supplementary Table S3. Gene-specific primers used for real-time RT-PCR assays in planktonic and sessile cells.

Target gene	Primer sequences 5′-3′	Taken from	Target genes	Primer sequences 5′-3′	Taken from
ACT	**F′** GATTTTGTCTGAACGTGGTAACAG<br>**R′** GGAGTTGAAAGTGGTTTGGTCAATAC	[1]	*EGF1*	**F′** TTGAGATGTTGCGGCAGGATA<br>**R′** ACTGGACAGACAGCAGGAC	[2]
18S rRNA	**F′** CACGACGGAGTTTCACAAGA<br>**R′** CGATGGAAGTTTGAGGCAAT	[3]	*ADH5*	**F′** ACCTGCAAGGGCTCATTCTG<br>**R′** CGGCTCTCAACTTCTCCATA	[2]
GAPDH	**F′** CGGTCCATCCCACAAGGA<br>**R′**AGTGGAAGATGGGATAATGTTACCA	[4]	*CSH1*	**F′** CGTGAGGACGAGAGAGAAT<br>**R′** CGAATGGACGACACAAAACA	[2]
KRE6	**F′** TCCAACAAGCATTATCAGCA<br>**R′** ATCACCAACAAACCATCGT	[5]	*GSC1*	**F′** CCCATTCTCTAGGCACGA<br>**R′** ATCAACAACCACTTGCTTCG	[2]
ALS3	**F′** GGTTATCGTCCATTTGTTGA<br>**R′** TTCTGTATCCAGTCCATCTT	[2]	*ZAP1*	**F′** ATCTGTCCAGTGTTGTTTGTA<br>**R′** AGGTCTCTTTGAAAGTTGTG	[2]
CYR1	**F′** GTTTCCCCCACCACTCA<br>**R′** TTGCGGTAATGACACAACAGA	[2]			

A. **B.**

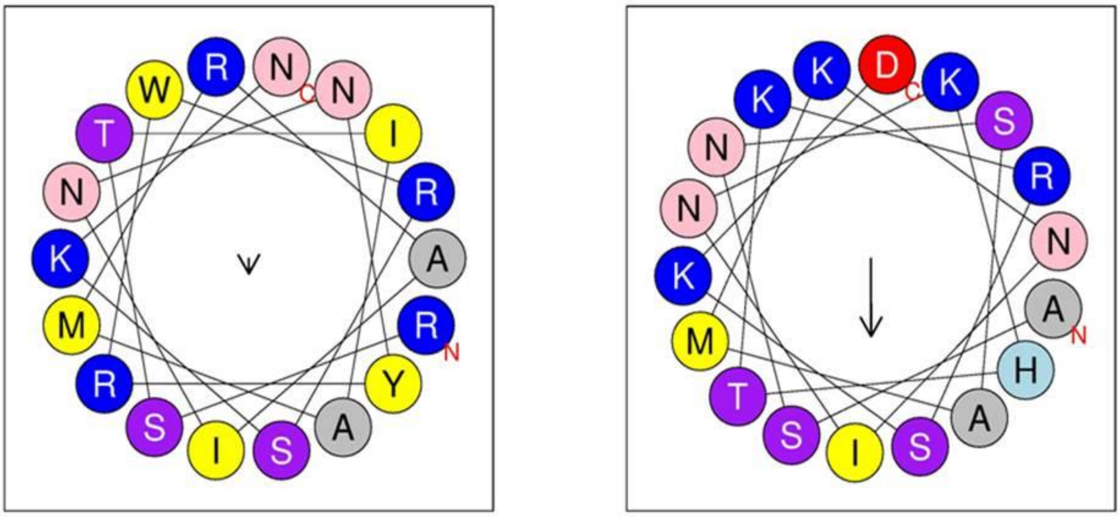

Supplementary Figure S1: Helical wheels of RNase 3(A) and RNase 7(B) 1-45mer N-termini peptides. A unique alpha–helix encompassing residues 22 to 40 has been considered as predicted by RMN for RNase 3 (drawn by heliquest [6]). Cationic residues are highlighted in blue, uncharged residues in grey, polar residues in purple, nonpolar residues in yellow, polar/uncharged residues in pink and anionic residues in red. N- terminal and C-terminal domains are designated with N and C letters respectively. Taken from [7].

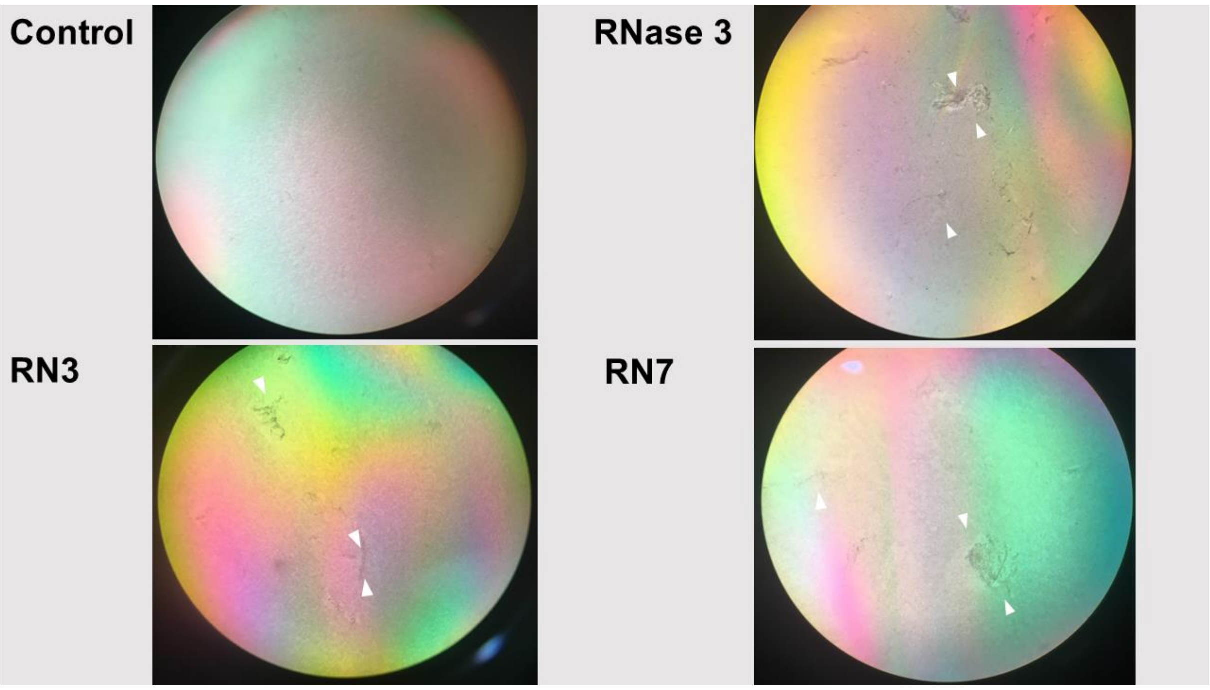

Supplementary Figure S2: Visualization of aggregation of *C. albicans* culture by RNase3, RN3 and RN7 peptides. Images were taken after 1 hour of incubation of *C. albicans* with 1 μM of RNase3, RN3 and RN7, as detailed in the Materials and Methods section, using a 50× stereoscope microscope.

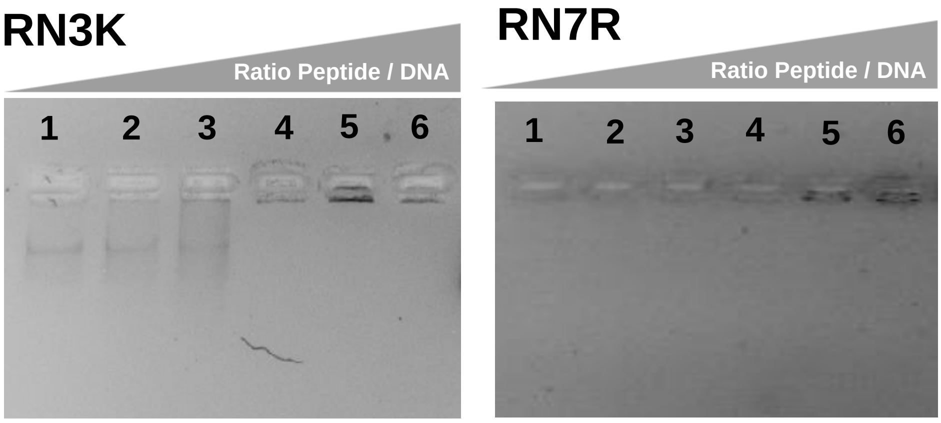

Supplementary Figure S3: Analysis of DNA binding activity of RN3 and RN7 substitute analogs using a gel retardation assay. The affinity to bind DNA was assayed by the inhibitory effect of the peptides on migration of DNA. Different amounts of the peptides were incubated with 200 ng of pET 28 plasmid DNA in 10 μL of binding buffer at room temperature during 20 min and subjected to electrophoresis on a 1.0% agarose gel. The first lane corresponds to negative control without peptide and the following lanes correspond to ratios peptide/DNA 2.5:1, 5:1, 10:1, 20:1 and 50:1 respectively.

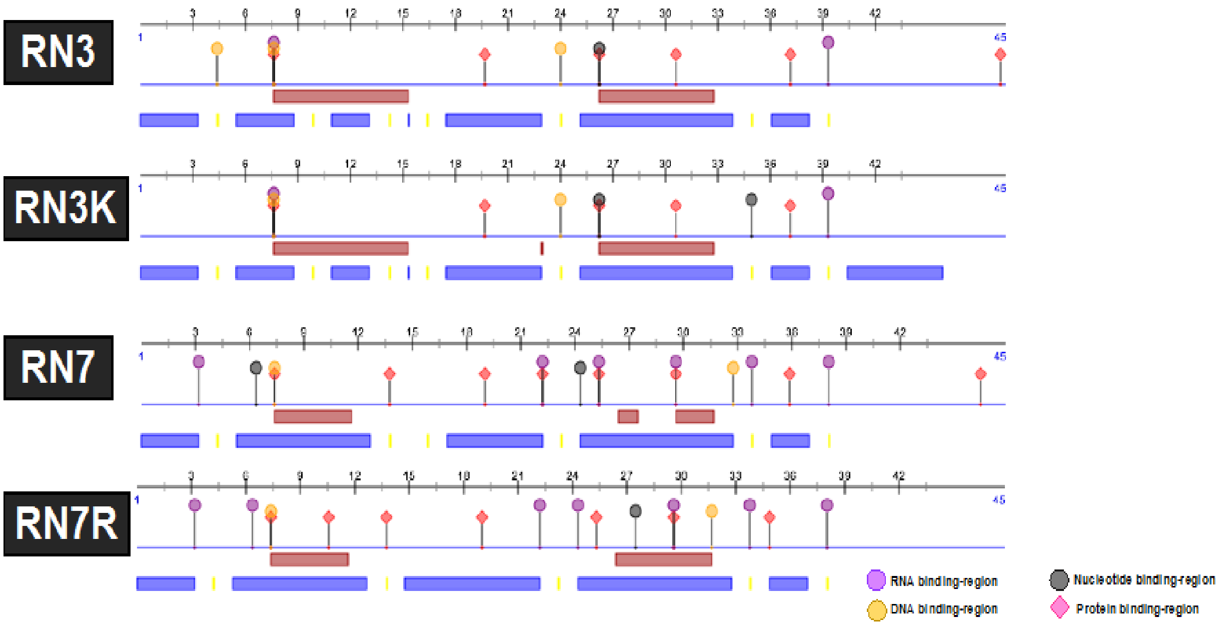

PEPTIDE (Secondary structure)	HELIX	STRAND	LOOP
	% in the protein		
RN1	37.50	4.17	58.33
RN2	44.44	4.44	51.11
RN3	44.44	0.0	55.56
RN3K	44.44	0.0	55.56
RN4	40.43	4.26	55.32
RN5	42.55	4.26	53.19
RN6	44.44	0.0	55.56
RN7	44.4	0.0	55.56
RN7R	42.22	0.0	57.78
RN8	44.44	0.0	55.56

Supplementary Figure S4: Secondary structure prediction of hRNase derived peptides (RN1-8) and K/R variants. The alpha-helix structure are represented by red horizontal line. The RNA or DNA binding residues are showed by circles or diamond forms. Percentage of helix, strand or loop structures are displayed in the embedded table. The figure was created using the online PredictProtein server: https://www.predictprotein.org.

Sample Information

Sample ID: RNAse1
Tray#: 1
Vail#: 31
Injection Volume: 60 uL
Method Filename: 5a60en15 '.lcm

Purity: 99 %

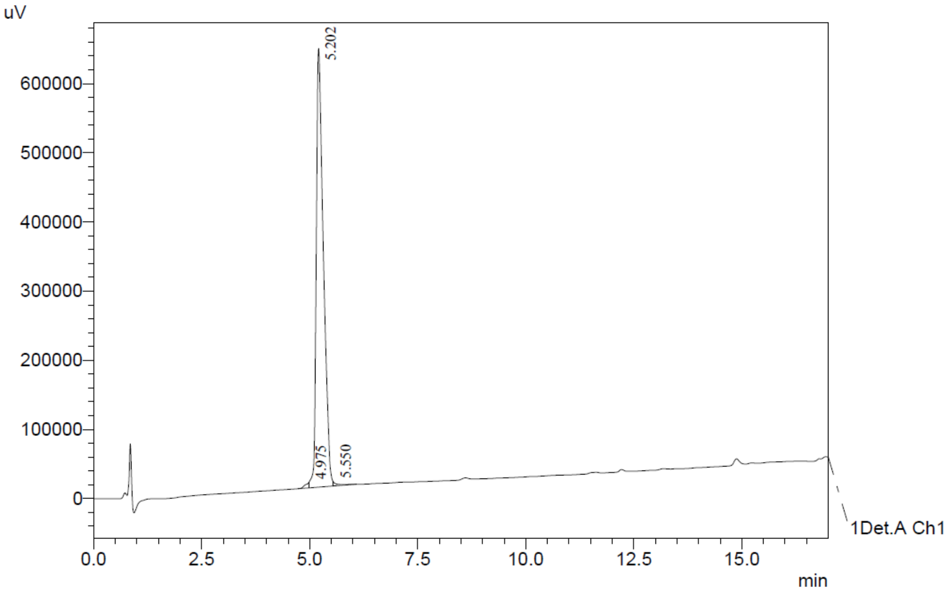

1 Det.A Ch1 / 220nm

PeakTable

Detector A Ch1 220nm

Peak#	Ret. Time	Area	Height	Area %
1	4.975	47048	7460	0.643
2	5.202	7229668	634292	98.774
3	5.550	42708	5638	0.583
Total		7319423	647390	100.000

Sample Information

Sample ID: RNAse2
Tray#: 1
Vail#: 31
Injection Volume: 10 uL
Method Filename: 20a60en15.lcm

Purity: 94 %

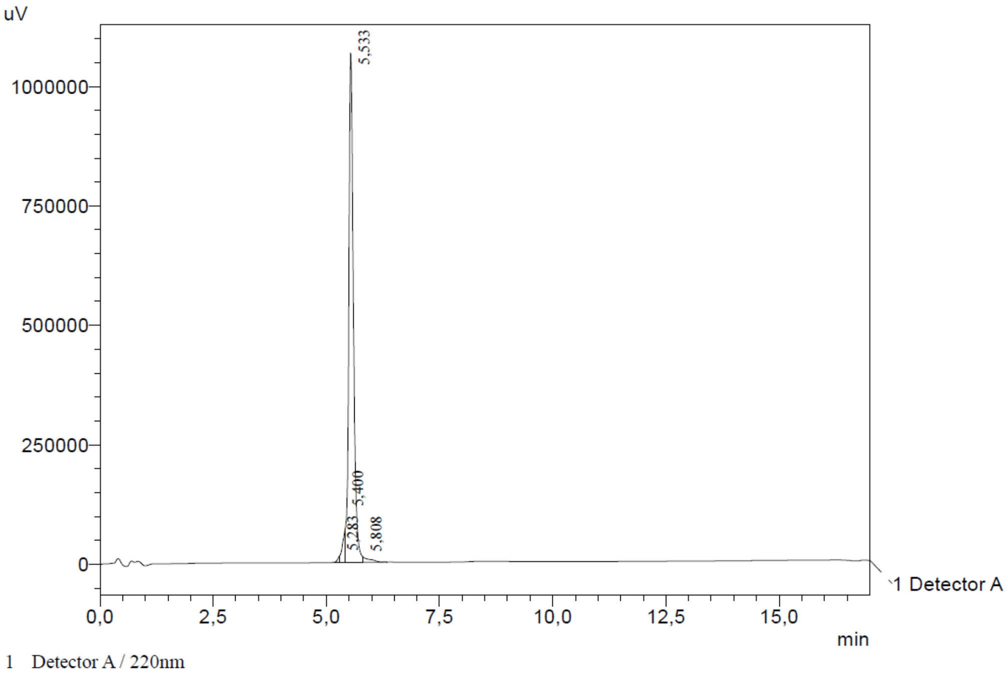

1 Detector A / 220nm

PeakTable

Detector A 220nm

Peak#	Ret. Time	Area	Height	Area %
1	5,283	40416	12570	0,502
2	5,400	260866	60736	3,241
3	5,533	7630691	1066270	94,790
4	5,808	118111	10881	1,467
Total		8050085	1150457	100,000

Sample Information

Sample ID: RNAse3
Tray#: 1
Vail#: 31
Injection Volume: 10 uL
Method Filename: 20a60en15.lcm

Purity: 98 %

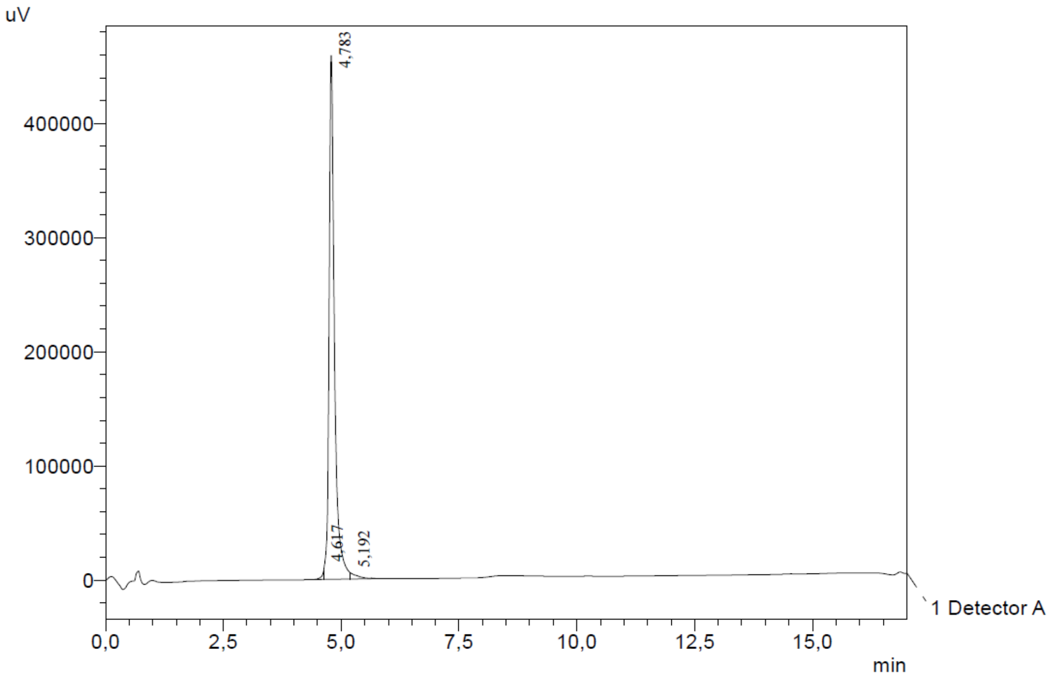

1 Detector A / 220nm

PeakTable

Detector A 220nm

Peak#	Ret. Time	Area	Height	Area %
1	4,617	20160	5591	0,539
2	4,783	3660667	458525	97,867
3	5,192	59625	5612	1,594
Total		3740453	469728	100,000

Sample Information

Sample ID: 3K
Tray#: 1
Vail#: 31
Injection Volume: 10 uL
Method Filename: 10a60en15.lcm

Purity: 90 %

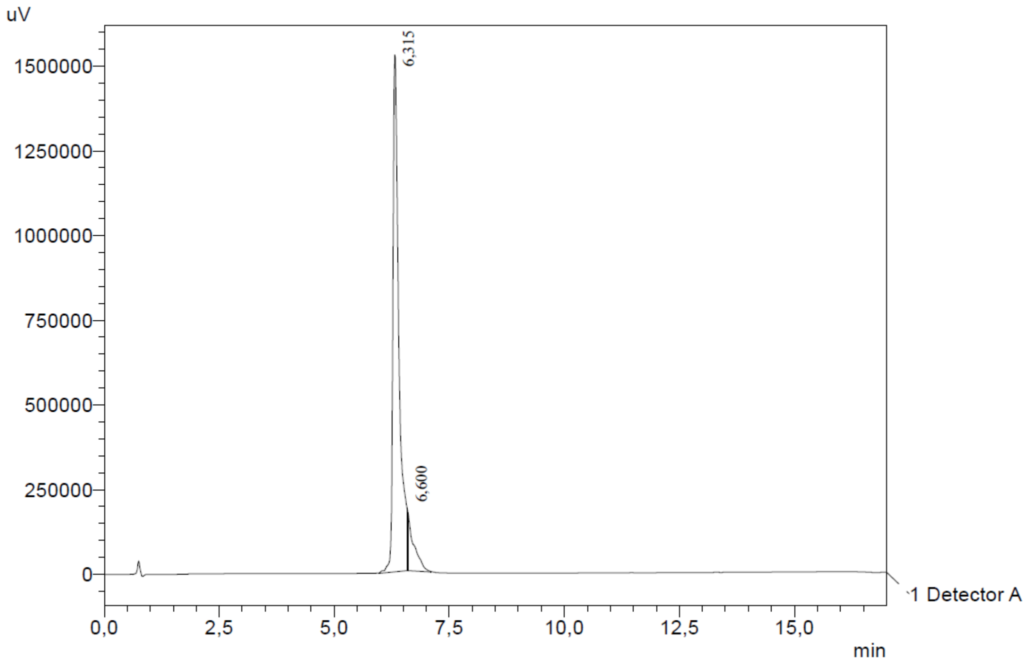

1 Detector A / 220nm

PeakTable

Detector A 220nm

Peak#	Ret. Time	Area	Height	Area %
1	6,315	13927245	1527024	90,500
2	6,600	1461988	171094	9,500
Total		15389233	1698118	100,000

Sample Information

Sample ID: RNAse4
Tray#: 1
Vail#: 31
Injection Volume: 15 uL
Method Filename: 15a50en15.lcm

Purity: 94 %

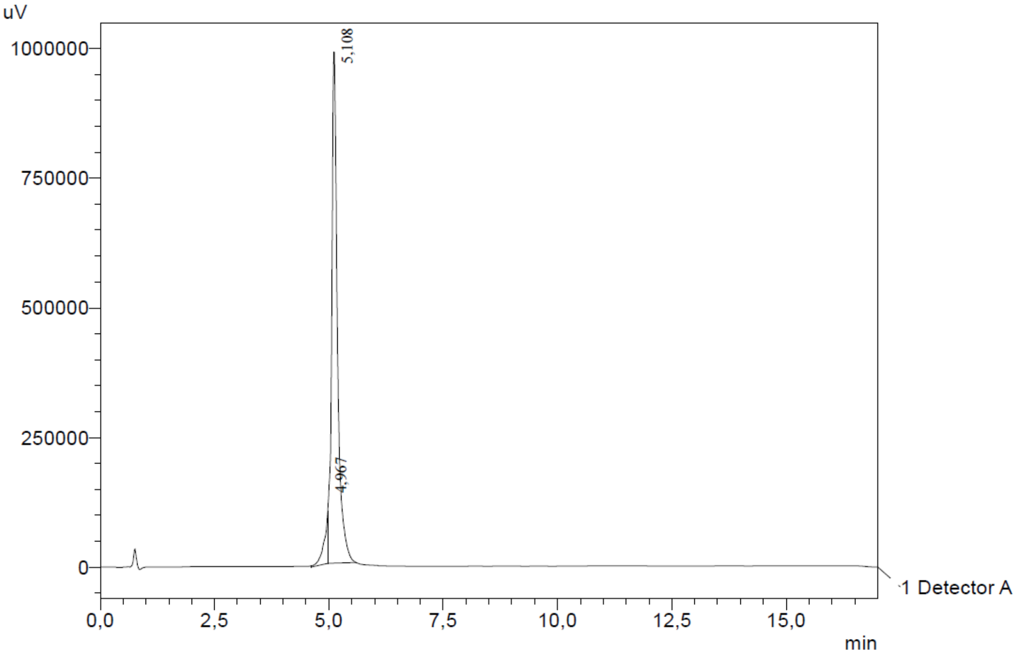

1 Detector A / 220nm

PeakTable

Detector A 220nm

Peak#	Ret. Time	Area	Height	Area %
1	4,967	550487	87732	5,940
2	5,108	8716228	986079	94,060
Total		9266715	1073810	100,000

Sample Information

Sample ID: RNAse5
Tray#: 1
Vail#: 31
Injection Volume: 15 uL
Method Filename: 10a50en15.lcm

Purity: 93 %

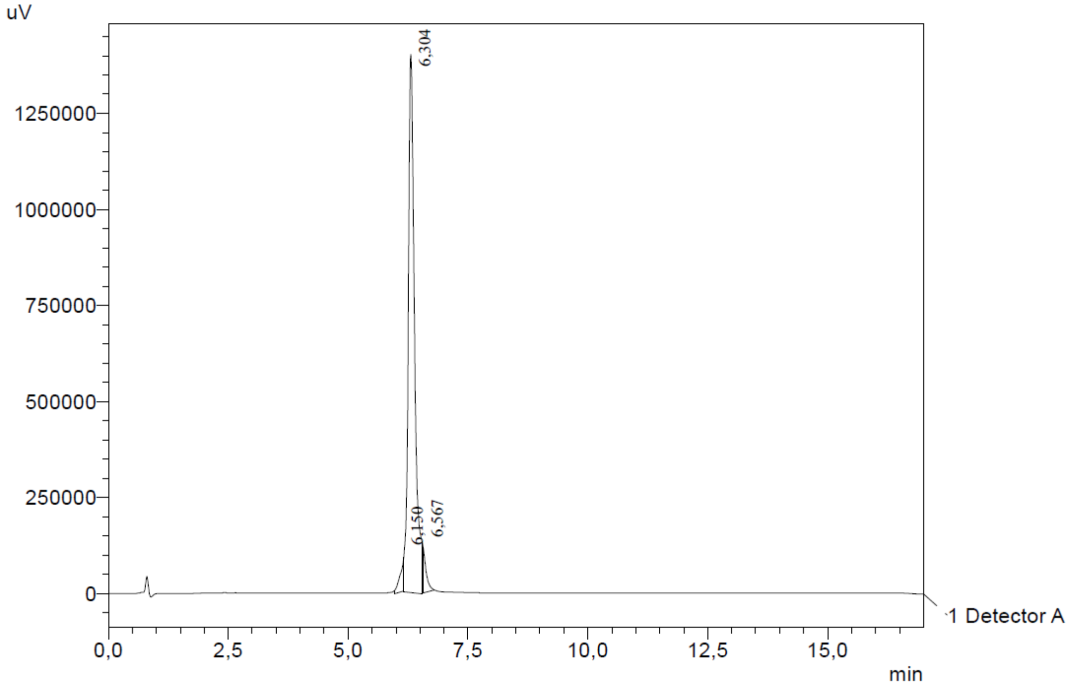

1 Detector A / 220nm

PeakTable

Detector A 220nm

Peak#	Ret. Time	Area	Height	Area %
1	6,150	450102	77985	3,279
2	6,304	12819865	1402122	93,407
3	6,567	454800	118479	3,314
Total		13724767	1598586	100,000

Sample Information

Sample ID: RNAse6
Tray#: 1
Vail#: 31
Injection Volume: 10 uL
Method Filename: 10a50en15.lcm

Purity: 99 %

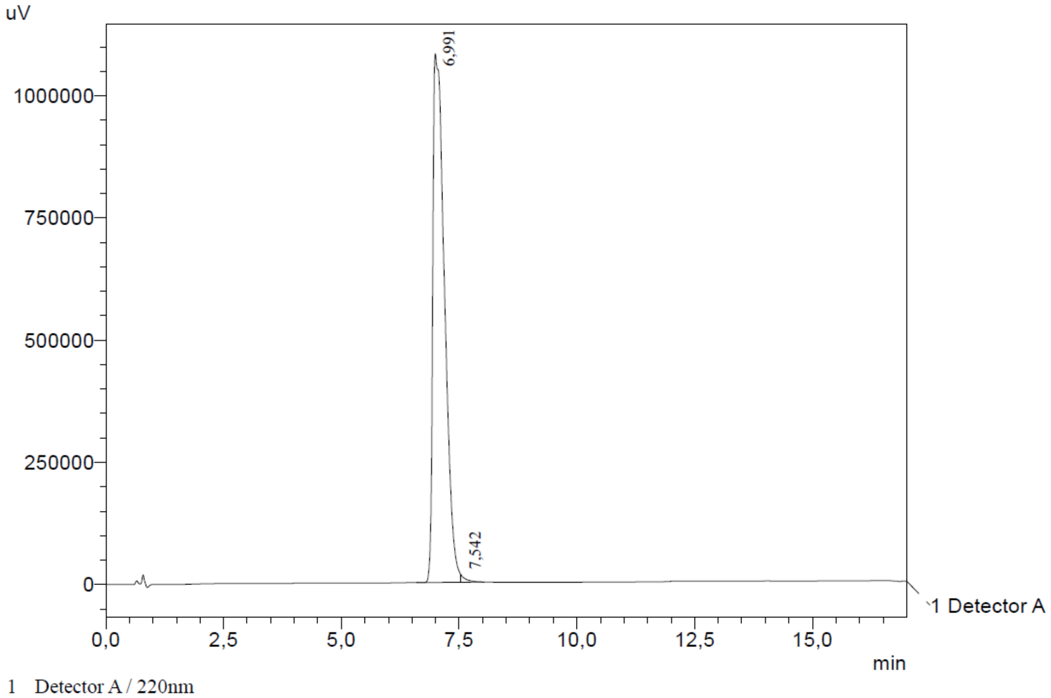

1 Detector A / 220nm

PeakTable

Detector A 220nm

Peak#	Ret. Time	Area	Height	Area %
1	6,991	18683708	1081656	99,457
2	7,542	101965	14456	0,543
Total		18785673	1096112	100,000

Sample Information

Sample ID: RNAse7
Tray#: 1
Vail#: 31
Injection Volume: 15 uL
Method Filename: 5a50en15.lcm

Purity: 96 %

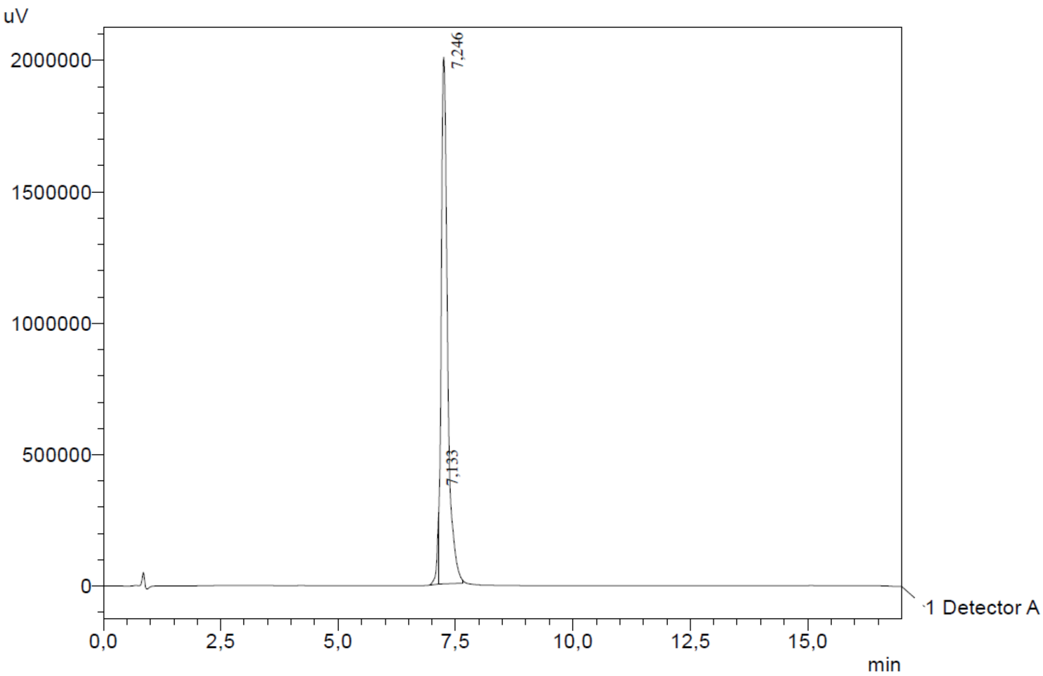

1 Detector A / 220nm

PeakTable

Detector A 220nm

Peak#	Ret. Time	Area	Height	Area %
1	7,133	723669	239353	3,738
2	7,246	18635881	2005087	96,262
Total		19359549	2244440	100,000

Sample Information

Sample ID: 7R
Tray#: 1
Vail#: 31
Injection Volume: 10 uL
Method Filename: 5a60en15.lcm

Purity: 90 %

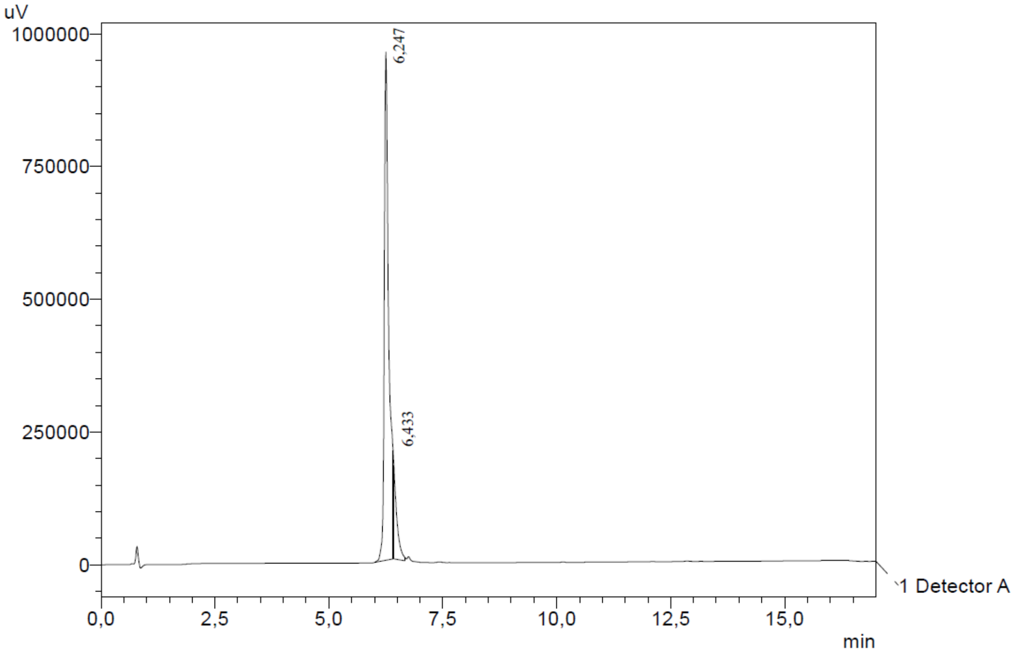

1 Detector A / 220nm

PeakTable

Detector A 220nm

Peak#	Ret. Time	Area	Height	Area %
1	6,247	6640126	957810	90,005
2	6,433	737418	161063	9,995
Total		7377544	1118873	100,000

Sample Information

Sample ID: RNAse8
Tray#: 1
Vail#: 31
Injection Volume: 15 uL
Method Filename: 10a50en15.lcm

Purity: 91 %

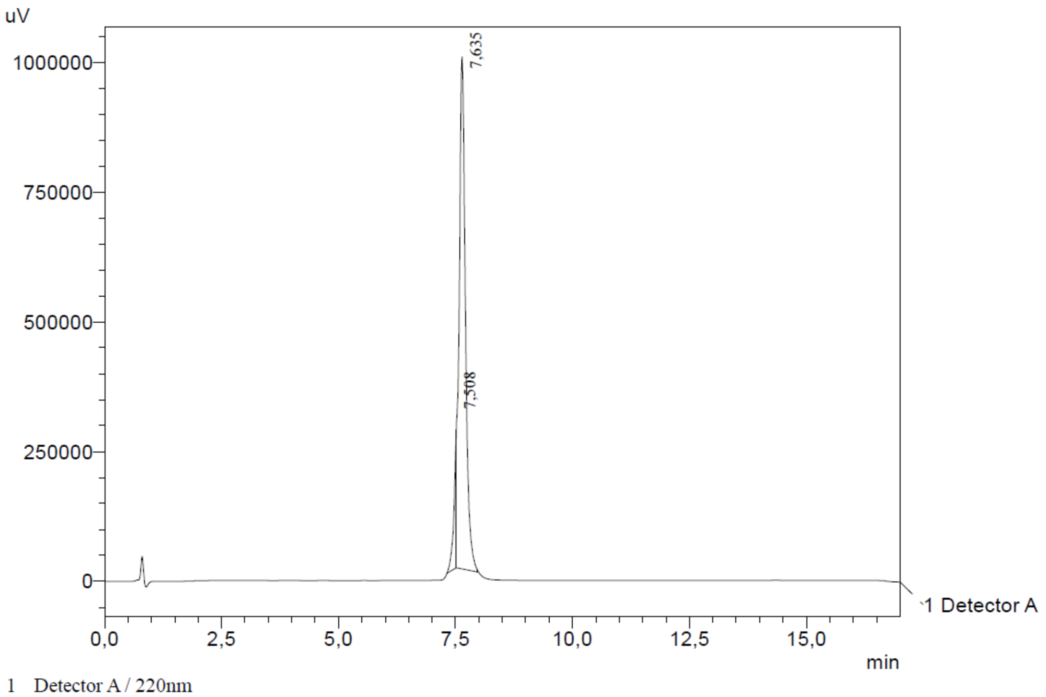

1 Detector A / 220nm

PeakTable

Detector A 220nm

Peak#	Ret. Time	Area	Height	Area %
1	7,508	931358	245038	9,155
2	7,635	9241640	987297	90,845
Total		10172998	1232335	100,000

Supplementary Figure S5. High Performance Liquid Chromatography (HPLC) profiles of purified RN peptides.

RN1

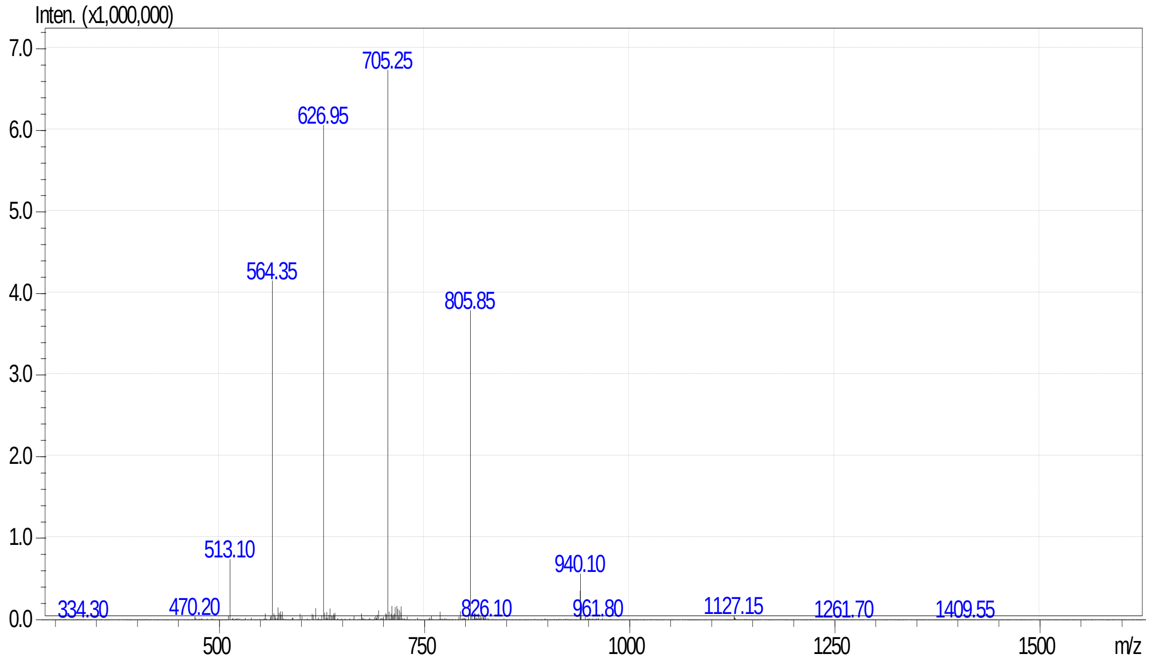

Positively charged ion series

Ch.	Average	MonoIso.
MH1+	5633.3180	5629.7241
MH2+	2817.1626	2815.3657
MH3+	1878.4442	1877.2462
MH4+	1409.0850	1408.1865
MH5+	1127.4694	1126.7506
MH6+	939.7257	939.1267
MH7+	805.6231	805.1097
MH8+	705.0461	704.5969
MH9+	626.8196	626.4203
MH10+	564.2383	563.8790
MH11+	513.0355	512.7088
MH12+	470.3665	470.0670
MH13+	434.2619	433.9855
MH14+	403.3152	403.0585
MH15+	376.4947	376.2551

RN2

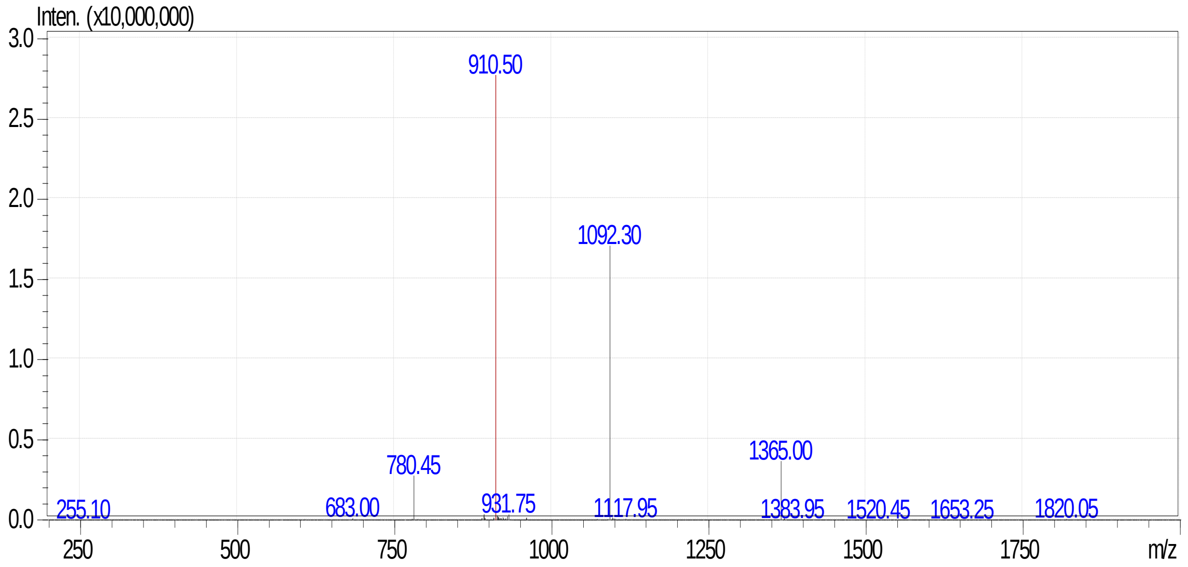

Positively charged ion series

Ch.	Average	MonoIso.
MH1+	5457.1367	5453.6807
MH2+	2729.0720	2727.3440
MH3+	1819.7171	1818.5651
MH4+	1365.0396	1364.1756
MH5+	1092.2332	1091.5420
MH6+	910.3622	909.7862
MH7+	780.4543	779.9606
MH8+	683.0235	682.5915
MH9+	607.2439	606.8599
MH10+	546.6202	546.2746
MH11+	497.0190	496.7049
MH12+	455.6847	455.3967
MH13+	420.7095	420.4437
MH14+	390.7308	390.4840
MH15+	364.7492	364.5188

RN3

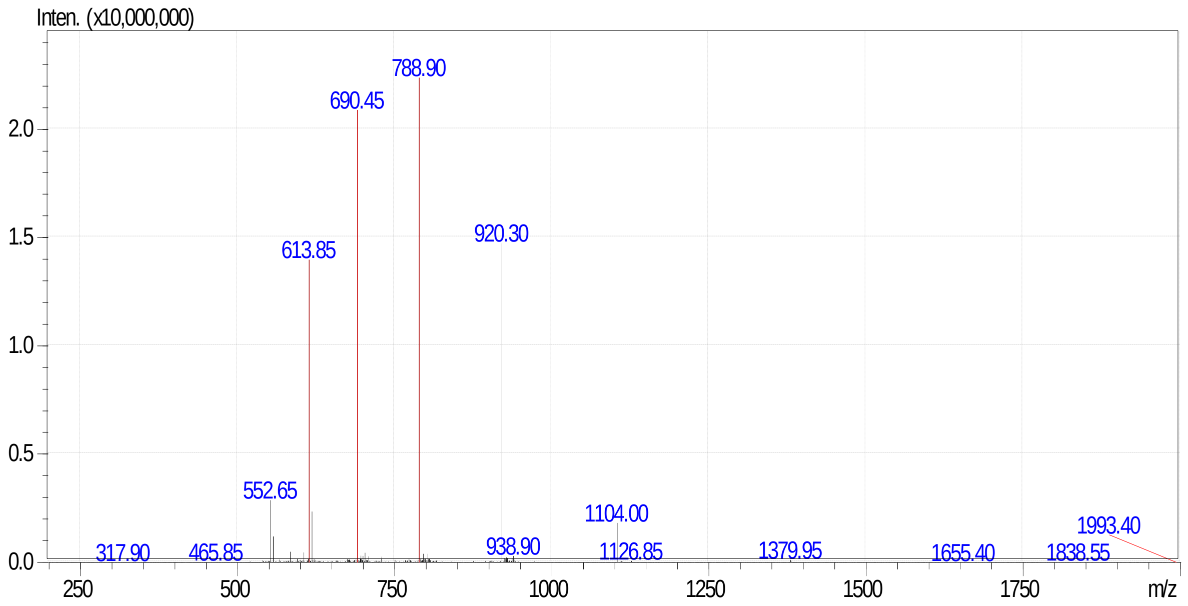

Positively charged ion series

Ch.	Average	MonoIso.
MH1+	5516.4596	5512.8694
MH2+	2758.7334	2756.9384
MH3+	1839.4914	1838.2947
MH4+	1379.8704	1378.9728
MH5+	1104.0977	1103.3797
MH6+	920.2493	919.6510
MH7+	788.9290	788.4162
MH8+	690.4388	689.9900
MH9+	613.8353	613.4364
MH10+	552.5525	552.1935
MH11+	502.4120	502.0857
MH12+	460.6283	460.3291
MH13+	425.2728	424.9967
MH14+	394.9682	394.7117
MH15+	368.7041	368.4648

RN3K

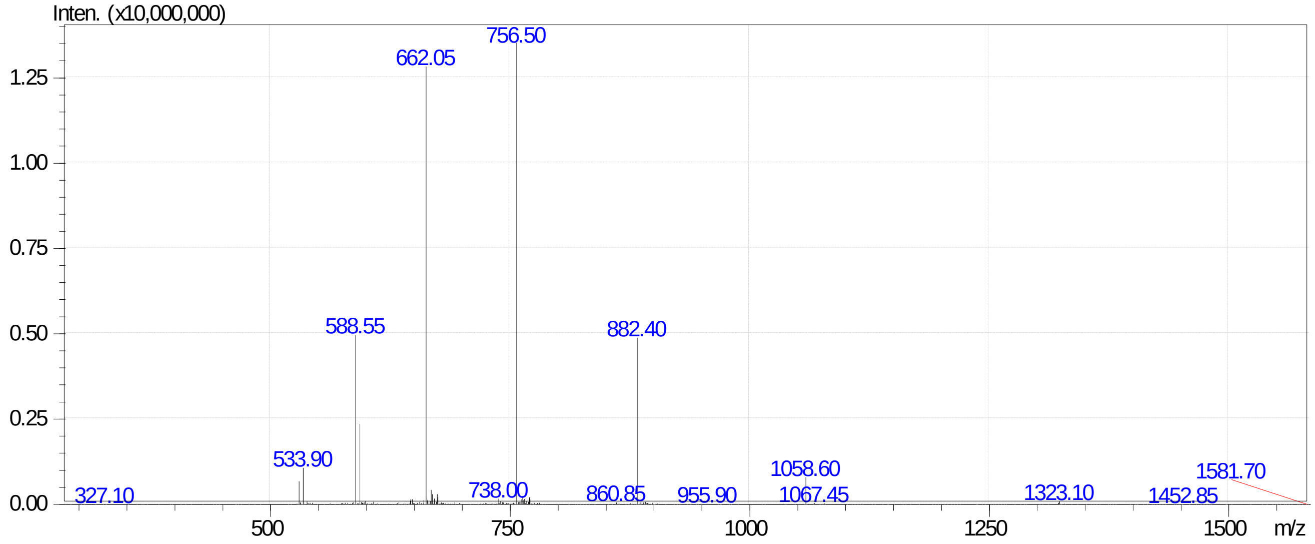

Positively charged ion series

Ch.	Average	MonoIso.
MH1+	5288.2326	5284.8721
MH2+	2644.6199	2642.9397
MH3+	1763.4157	1762.2955
MH4+	1322.8136	1321.9735
MH5+	1058.4523	1057.7802
MH6+	882.2115	881.6514
MH7+	756.3252	755.8451
MH8+	661.9104	661.4904
MH9+	588.4768	588.1034
MH10+	529.7298	529.3938
MH11+	481.6641	481.3586
MH12+	441.6094	441.3293
MH13+	407.7169	407.4584
MH14+	378.6662	378.4262
MH15+	353.4890	353.2649

RN4

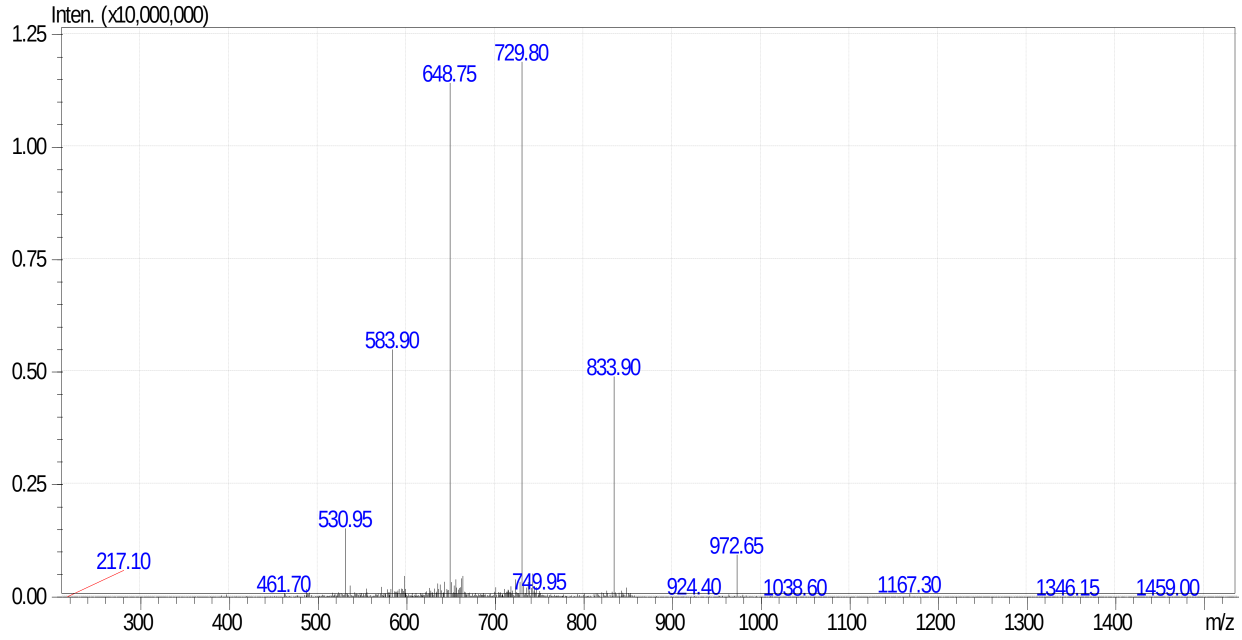

Positively charged ion series

Ch.	Average	MonoIso.
MH1+	5829.6583	5825.8434
MH2+	2915.3328	2913.4254
MH3+	1943.8909	1942.6193
MH4+	1458.1700	1457.2163
MH5+	1166.7375	1165.9745
MH6+	972.4491	971.8133
MH7+	833.6717	833.1267
MH8+	729.5887	729.1118
MH9+	648.6352	648.2113
MH10+	583.8724	583.4909
MH11+	530.8846	530.5378
MH12+	486.7282	486.4103
MH13+	449.3650	449.0716
MH14+	417.3395	417.0670
MH15+	389.5840	389.3297

RN5

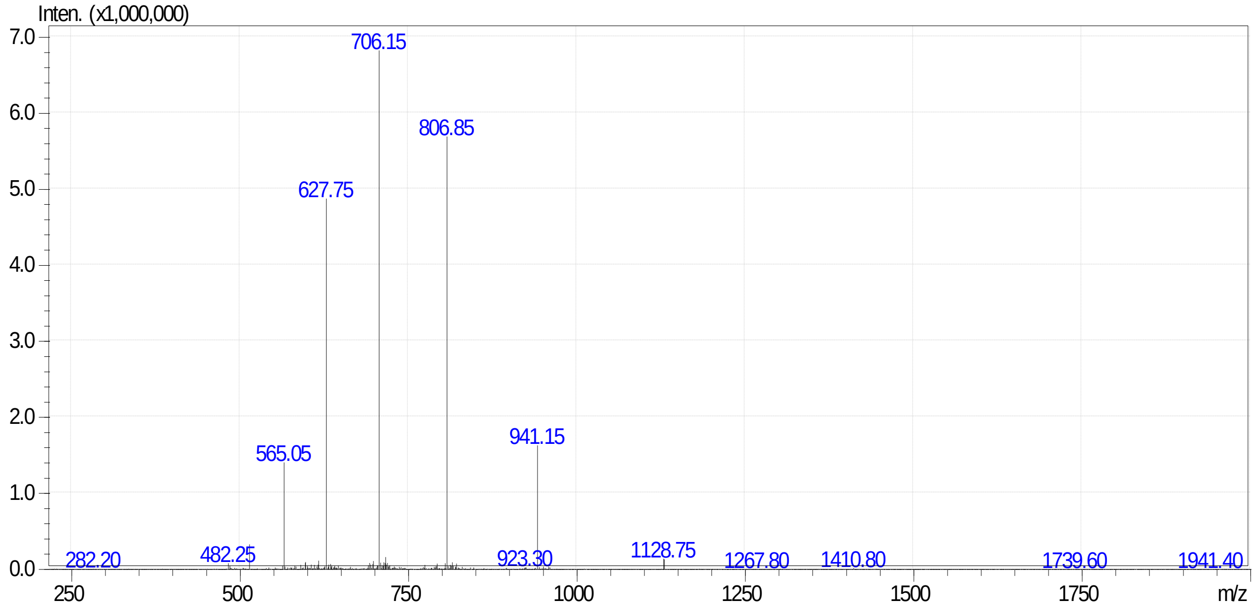

Positively charged ion series

Ch.	Average	MonoIso.
MH1+	5640.1965	5636.7701
MH2+	2820.6019	2818.8887
MH3+	1880.7370	1879.5949
MH4+	1410.8046	1409.9480
MH5+	1128.8451	1128.1598
MH6+	940.8721	940.3011
MH7+	806.6057	806.1163
MH8+	705.9059	705.4776
MH9+	627.5839	627.2031
MH10+	564.9262	564.5836
MH11+	513.6608	513.3494
MH12+	470.9397	470.6542
MH13+	434.7911	434.5275
MH14+	403.8065	403.5618
MH15+	376.9532	376.7248

RN6

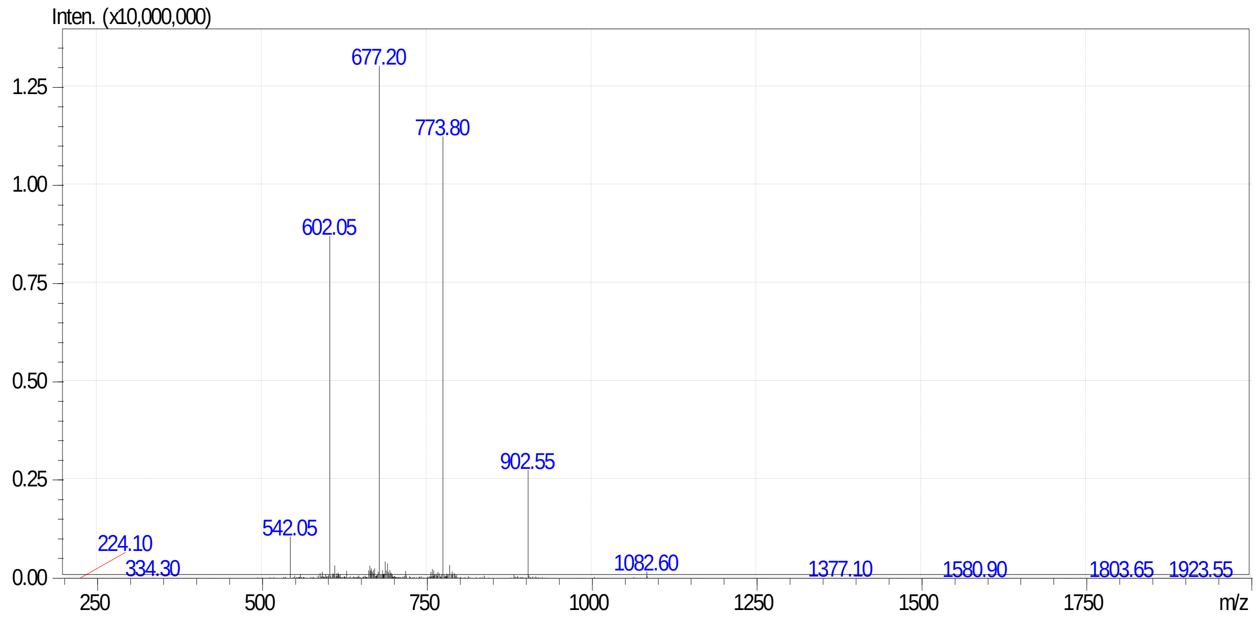

Positively charged ion series

Ch.	Average	MonoIso.
MH1+	5409.1231	5405.7515
MH2+	2705.0652	2703.3794
MH3+	1803.7126	1802.5887
MH4+	1353.0362	1352.1933
MH5+	1082.6304	1081.9561
MH6+	902.3599	901.7980
MH7+	773.5953	773.1136
MH8+	677.0218	676.6003
MH9+	601.9090	601.5344
MH10+	541.8189	541.4817
MH11+	492.6542	492.3477
MH12+	451.6836	451.4026
MH13+	417.0162	416.7568
MH14+	387.3013	387.0604
MH15+	361.5483	361.3236

RN7

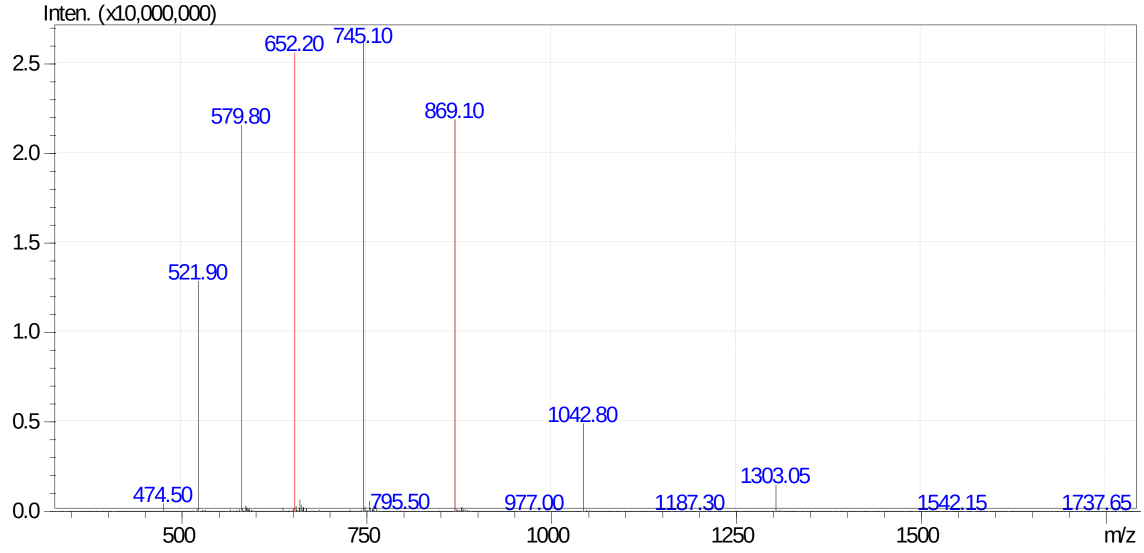

Positively charged ion series

Ch.	Average	MonoIso.
MH1+	5210.0377	5206.6724
MH2+	2605.5225	2603.8399
MH3+	1737.3507	1736.2290
MH4+	1303.2649	1302.4236
MH5+	1042.8134	1042.1403
MH6+	869.1790	868.6181
MH7+	745.1545	744.6737
MH8+	652.1361	651.7154
MH9+	579.7884	579.4145
MH10+	521.9103	521.5738
MH11+	474.5555	474.2496
MH12+	435.0931	434.8127
MH13+	401.7019	401.4431
MH14+	373.0809	372.8405
MH15+	348.2760	348.0516

RN7R

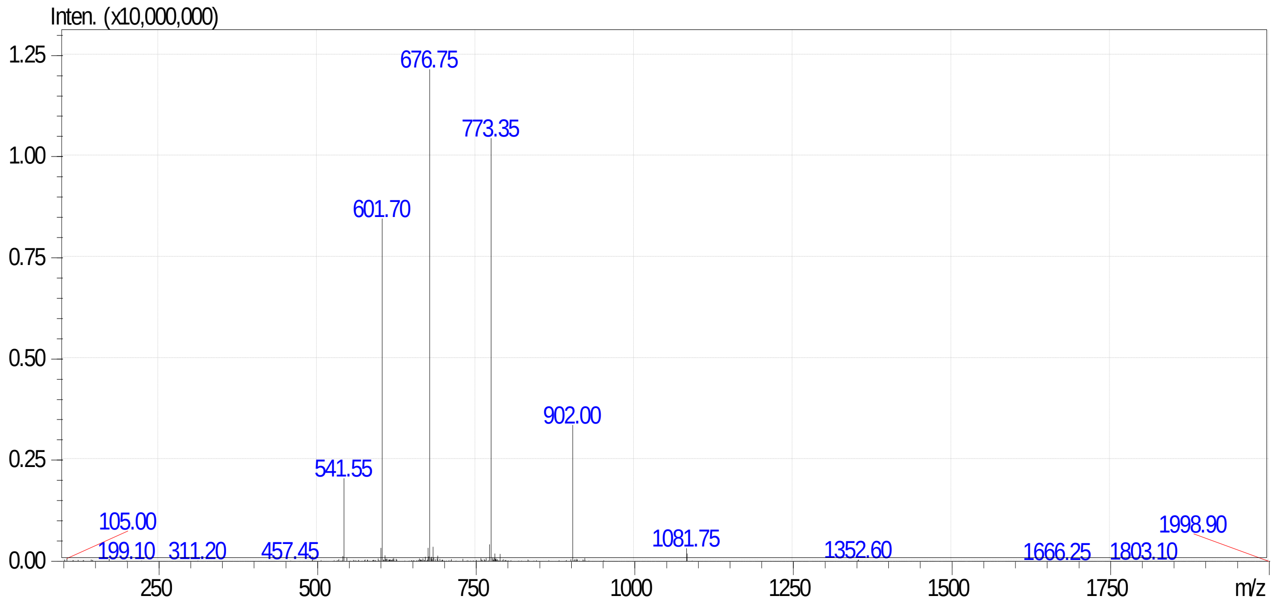

Positively charged ion series

Ch.	Average	MonoIso.
MH1+	5406.1315	5402.7155
MH2+	2703.5694	2701.8614
MH3+	1802.7153	1801.5767
MH4+	1352.2883	1351.4343
MH5+	1082.0321	1081.3489
MH6+	901.8613	901.2920
MH7+	773.1679	772.6799
MH8+	676.6478	676.2208
MH9+	601.5766	601.1971
MH10+	541.5197	541.1781
MH11+	492.3822	492.0717
MH12+	451.4343	451.1496
MH13+	416.7861	416.5233
MH14+	387.0876	386.8436
MH15+	361.3489	361.1212

RN8

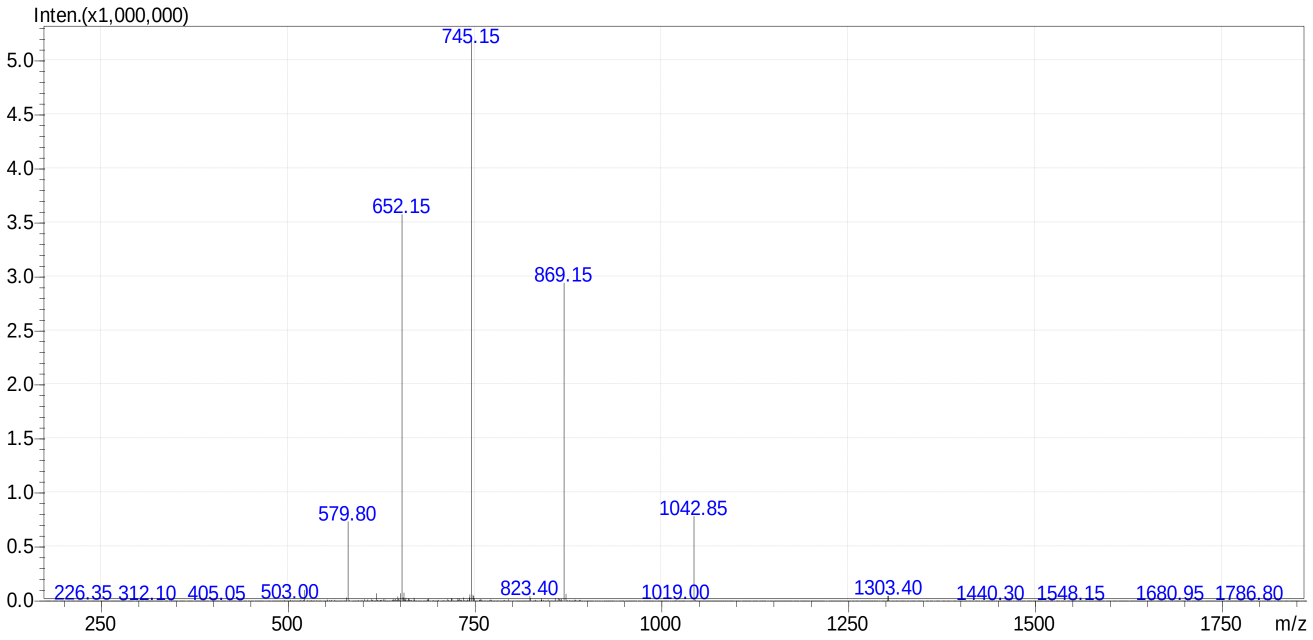

Positively charged ion series

Ch.	Average	MonoIso.
MH1+	5208.8900	5205.5997
MH2+	2604.9486	2603.3035
MH3+	1736.9682	1735.8714
MH4+	1302.9780	1302.1554
MH5+	1042.5838	1041.9258
MH6+	868.9877	868.4393
MH7+	744.9905	744.5205
MH8+	651.9926	651.5813
MH9+	579.6609	579.2953
MH10+	521.7955	521.4665
MH11+	474.4512	474.1520
MH12+	434.9975	434.7233
MH13+	401.6136	401.3605
MH14+	372.9989	372.7639
MH15+	348.1995	347.9801

Supplementary Figure S6. Liquid chromatography- Mass Spectrometry (LCMS) spectra of purified RN peptides.

References

1. Naglik, J.R.; Fostira, F.; Ruprai, J.; Staab, J.F.; Challacombe, S.J.; Sundstrom, P. Candida albicans HWP1 gene expression and host antibody responses in colonization and disease. *J. Med. Microbiol.* **2006**, *55*, 1323–1327.

2. Morici, P.; Fais, R.; Rizzato, C.; Tavanti, A.; Lupetti, A. Inhibition of Candida albicans biofilm formation by the synthetic lactoferricin derived peptide hLF1-11. *PLoS One* **2016**, *11*, 1–15.

3. Liu, T.T.; Lee, R.E.B.; Barker, K.S.; Lee, R.E.; Wei, L.; Homayouni, R.; Rogers, P.D. Genome-wide expression profiling of the response to azole, polyene, echinocandin, and pyrimidine antifungal agents in Candida albicans. *Antimicrob. Agents Chemother.* **2005**, *49*, 2226–2236.

4. Nailis, H.; Coenye, T.; Van Nieuwerburgh, F.; Deforce, D.; Nelis, H.J. Development and evaluation of different normalization strategies for gene expression studies in Candida albicans biofilms by real-time PCR. *BMC Mol. Biol.* **2006**, *7*, 1–9.

5. Tsai, P.W.; Cheng, Y.L.; Hsieh, W.P.; Lan, C.Y. Responses of Candida albicans to the human antimicrobial peptide LL-37. *J. Microbiol.* **2014**, *52*, 581–589.

6. Gautier, R.; Douguet, D.; Antonny, B.; Drin, G. HELIQUEST: a web server to screen sequences with specific -helical properties. *Bioinformatics* **2008**, *24*, 2101–2102.

7. García-Mayoral, M.F.; Moussaoui, M.; de la Torre, B.G.; Andreu, D.; Boix, E.; Nogués, M.V.; Rico, M.; Laurents, D. V; Bruix, M. NMR structural determinants of eosinophil cationic protein binding to membrane and heparin mimetics. *Biophys. J.* **2010**, *98*, 2702–11.
